# The Inner Membrane Protein PilG Interacts with DNA and the Secretin PilQ in Transformation

**DOI:** 10.1371/journal.pone.0134954

**Published:** 2015-08-06

**Authors:** Stephan A. Frye, Emma Lång, Getachew Tesfaye Beyene, Seetha V. Balasingham, Håvard Homberset, Alexander D. Rowe, Ole Herman Ambur, Tone Tønjum

**Affiliations:** 1 Department of Microbiology, Oslo University Hospital, Oslo, Norway; 2 Department of Microbiology, University of Oslo, Oslo, Norway; Centre National de la Recherche Scientifique, Aix-Marseille Université, FRANCE

## Abstract

Expression of type IV pili (Tfp), filamentous appendages emanating from the bacterial surface, is indispensable for efficient neisserial transformation. Tfp pass through the secretin pore consisting of the membrane protein PilQ. PilG is a polytopic membrane protein, conserved in Gram-positive and Gram-negative bacteria, that is required for the biogenesis of neisserial Tfp. PilG null mutants are devoid of pili and non-competent for transformation. Here, recombinant full-length, truncated and mutated variants of meningococcal PilG were overexpressed, purified and characterized. We report that meningococcal PilG directly binds DNA *in vitro*, detected by both an electromobility shift analysis and a solid phase overlay assay. PilG DNA binding activity was independent of the presence of the consensus DNA uptake sequence. PilG-mediated DNA binding affinity was mapped to the N-terminus and was inactivated by mutation of residues 43 to 45. Notably, reduced meningococcal transformation of DNA *in vivo* was observed when PilG residues 43 to 45 were substituted by alanine *in situ*, defining a biologically significant DNA binding domain. N-terminal PilG also interacted with the N-terminal region of PilQ, which previously was shown to bind DNA. Collectively, these data suggest that PilG and PilQ in concert bind DNA during Tfp-mediated transformation.

## Introduction

*Neisseria meningitidis*, the meningococcus (Mc), is a human-specific opportunistic pathogen and one of the leading causative agents of meningitis and septicaemia worldwide [1].

Type IV pili (Tfp) are filamentous appendages emanating from the bacterial surface that are required for adherence of bacteria to human cells and for transformation of DNA [2, 3]. Biogenesis of Tfp is not well characterized, but several proteins required for pilus assembly, extrusion and retraction have been identified [4]. In *Neisseria sp*., these include PilE [5], ComP [6], the secretin PilQ [7–10], the lipoproteins PilP [11] and PilW [12], the prepilin peptidase PilD [13], the ATPase PilT, which is driving pilus retraction [14, 15], and the adhesin PilC [16]. Tfp pass through the outer membrane secretin pore consisting of the dodecamer PilQ [7, 9, 10, 17].

The neisserial Tfp biogenesis machinery is related to the type II secretion (T2S) system, suggesting that these pathways could function in similar ways [18]. At least 12 proteins are required for this secretion mechanism, which is broadly conserved among Gram-negative bacteria [18]. The GspF protein family includes a large number of inner membrane proteins engaged in T2S and Tfp biogenesis, including the neisserial protein PilG [19, 20]. PilG is a polytopic membrane protein, and the 3D-structure of a PilG multimer has been modeled using electron microscopy and single particle averaging [19]. PilG null mutants are devoid of pili and are not competent for DNA transformation [20]. PilG orthologs are found widely in Gram-positive and Gram-negative bacterial species [19, 21, 22]. Donnenberg and co-workers revealed that the PilG ortholog BfpE in enteropathogenic *Escherichia coli* (EPEC) plays a role in bundle-forming pili (BFP) retraction [23]. In that study, BFP retraction was dependent on the direct interaction between the cytoplasmic PilT-like ATPase BfpF and the N-terminus of BfpE. In *Pseudomonas aeruginosa* the N-terminal cytoplasmic domain of the PilG-ortholog protein PilC interacts with the ATPase PilB and the C-terminal cytoplasmic domain of PilC probably with PilT [24]. Interestingly, BfpE is the first protein identified, in addition to PilT-like proteins, to play a role in pilus retraction. The EPEC secretin BfpB targets BFP to the surface [25], but it is not reported whether BfpE and BfpB interact. Nevertheless, the exact function of GspF homologs, including PilG, remains unknown.

The uptake of exogenous DNA into the Mc cell during transformation is a multi-step process that requires type IV pili [26]. Although little is known about Mc proteins involved in DNA binding and uptake [27, 28], the PilQ pore, which binds DNA [10], may play a role in this process. Competence factors ComL and ComE are also suggested to facilitate transport of incoming DNA through the periplasm [29–32] and ComA is thought to promote transport of DNA across the cytoplasmic membrane [33]. However, additional components may participate in the DNA binding and processing during transformation.

Neisserial DNA contains more than 2000 copies of the specific 10–12 bp DNA uptake sequence (DUS), which is preferentially taken up in neisserial transformation [34, 35]. Therefore, it has been proposed that neisserial cells express a DUS-specific receptor, and DUS specificity has been demonstrated for the neisserial ComP protein [36, 37]. Together with Tfp, DNA binding proteins and the PilQ pore directly facilitate DNA uptake.

We have previously shown that PilG is an inner membrane protein that binds DNA [38] and hypothesize that PilG is also involved in the non-specific binding and uptake of transforming DNA occurring in the wake of pilus retraction through the PilQ pore [10, 11].

The goal of this study was to characterize the role of Mc PilG in DNA binding and transformation. To this end, recombinant full-length and truncated PilG variants were overexpressed and purified. The DNA and protein binding properties of these recombinant proteins were analyzed *in vitro*, including their interactions with other Mc proteins as well as DNA with and without DUS. We report that N-terminal PilG exhibits intrinsic DUS-independent DNA binding activity and that PilG interacts with the secretin PilQ. Notably, reduced competence for transformation *in vivo* was observed when PilG residues of the putative DNA binding domain were substituted by alanine. These data suggest that PilG, in addition to its role in pilus biogenesis [20], has a functional DNA binding domain, and that it thus may play a dual role during transformation of Mc cells.

## Materials and Methods

### Strains, plasmids and constructs

Strains, plasmids and constructs employed in the study are listed in Table 1. *N*. *meningitidis* strains MC58 and M1080 were grown on blood agar plates in a 5% CO_2_ atmosphere at 37°C. *E*. *coli* strain ER2566 (New England Biolabs), used for plasmid propagation and recombinant protein expression, was grown in LB medium or on LB agar plates containing kanamycin (50 μg ml^-1^) at 37°C.

**Table 1:** Strains, plasmids and constructs employed in the study.

Strain or plasmid	Relevant characteristics	Reference or source
***N*. *meningitidis* strains**		
MC58	Serogroup B, isolated in the UK, 1983	[72]
M1080	Serogroup B, isolated in the USA, 1969	[73]
MC58 ΔpilG	*pilG* K.O. clone of MC58, erythromycin resistant	Clone GG1 in [20]
MC58-pilG:aph	MC58 transformed with pSAF67, carrying wild type *pilG* with *aph* inserted downstream	This study
MC58-pilG-EEE:aph	MC58 transformed with pSAF68B, carrying *pilG*_*EEE39-41AAA*_ with *aph* inserted downstream	This study
MC58-pilG-RKK:aph	MC58 transformed with pSAF69, carrying wild type *pilG*_*RKK43-45AAA*_ with *aph* inserted downstream	This study
M1080-pilG:aph	M1080 transformed with pSAF67, carrying wild type *pilG* with *aph* inserted downstream	This study
M1080-pilG-EEE:aph	M1080 transformed with pSAF68B, carrying *pilG*_*EEE39-41AAA*_ with *aph* inserted downstream	This study
M1080-pilG-RKK:aph	M1080 transformed with pSAF69, carrying wild type *pilG*_*RKK43-45AAA*_ with *aph* inserted downstream	This study
***E*. *coli* strain**		
ER2566	Expression strain with a chromosomal copy of the T7 RNA polymerase gene	New England Biolabs
**Plasmid/construct**		
pET28b(+)	Expression vector based on a T7 promoter-driven system, 6×His tag, Kan^r^	Novagen
pPilG1	pET28b(+) with PilG_FL/1-410_ insert	This study
pPilG-I	pET28b(+) with PilG_1-256_ insert	This study
pPilG-II	pET28b(+) with PilG_1-178_ insert	This study
pPilG-II:1	pET28b(+) with PilG_1-60_ insert	This study
pPilG-II:3	pET28b(+) with PilG_1-140_ insert	This study
pPilG-II:4–1	pET28b(+) with PilG_1-80_ insert	This study
pPilG-II:4–2	pET28b(+) with PilG_1-81_ insert	This study
pPilG-II:6–1	pET28b(+) with PilG_30-80_ insert	This study
pPilG-II:6–2	pET28b(+) with PilG_30-81_ insert	This study
pPilG-IV	pET28b(+) with PilG_257-410_ insert	This study
pPilG-V	pET28b(+) with PilG_166-410_ insert	This study
pPilG-K3A	pPilG1 with alanine substitution at aa 3	This study
pPilG-KK12-13AA	pPilG1 with alanine substitution at aa 12–13	This study
pPilG-E14A	pPilG1 with alanine substitution at aa 14	This study
pPilG-KR15-16AA	pPilG1 with alanine substitution at aa 15–16	This study
pPilG-EEE39-41AAA	pPilG1 with alanine substitution at aa 39–41	This study
pPilG-RKK43-45AAA	pPilG1 with alanine substitution at aa 43–45	This study
pPilG-E41H/RKK43-45AAA	pPilG1 with alanine substitution at aa 43–45 and change of aa 41 from E to H	This study
pPilG-Q55A	pPilG1 with alanine substitution at aa 55	This study
pPilG-SS62-63AA	pPilG1 with alanine substitution at aa 62–63	This study
pBluescript II SK(+)		Stratagene
pUP6	Tn5 *aph* containing plasmid	[6]
pOHA-D4	Variant of p0-DUS, containing AT-DUS	[34, 74]
pSAF67	pBluescript II SK(+) based plasmid containing wild type *pilG*, Kan^r^	This study
pSAF68B	pSAF67 derivative, *pilG*_*EEE39-41AAA*_, Kan^r^	This study
pSAF69	pSAF67 derivative, *pilG*_*RKK43-45AAA*_, Kan^r^	This study

### Bioinformatics analyses and search for signature motifs

Predictions on the meningococcal MC58 PilG (NP_273382) subcellular location was performed by using the PSORTb service [39], and the sequence was assessed for native disorder prediction by DISOPRED2 [40] and VSL1 [41]. Searches for functional domains or signature motifs in the PilG sequence were performed using the DOLOP [42], PROSITE [43] and Pfam databases [44]. Primary prediction of the PilG secondary structure and the prevalence of alpha-helical elements was performed by using the JPred service [45] and the PSIPRED service [46], while the presence and location of transmembrane helices was predicted by MEMSAT3 [47]. The presence of DNA binding motifs was first assessed by using the ExPASy site [48], whereas the electrostatic charge was calculated by using the charge program from the EMBOSS package [49]. In the search for DNA binding motifs, the PilG sequence was broken down into three parts, divided into the major groups of transmembrane helices and submitted separately to SAM-T06 for sequence-based fold recognition by use of a hidden Markov method [50]. Subsequently, the PilG sequence was submitted to the DP-Bind server [51] for additional prediction of sequence-based DNA binding sites.

### Cloning and overexpression of PilG constructs

**Fig 1:**
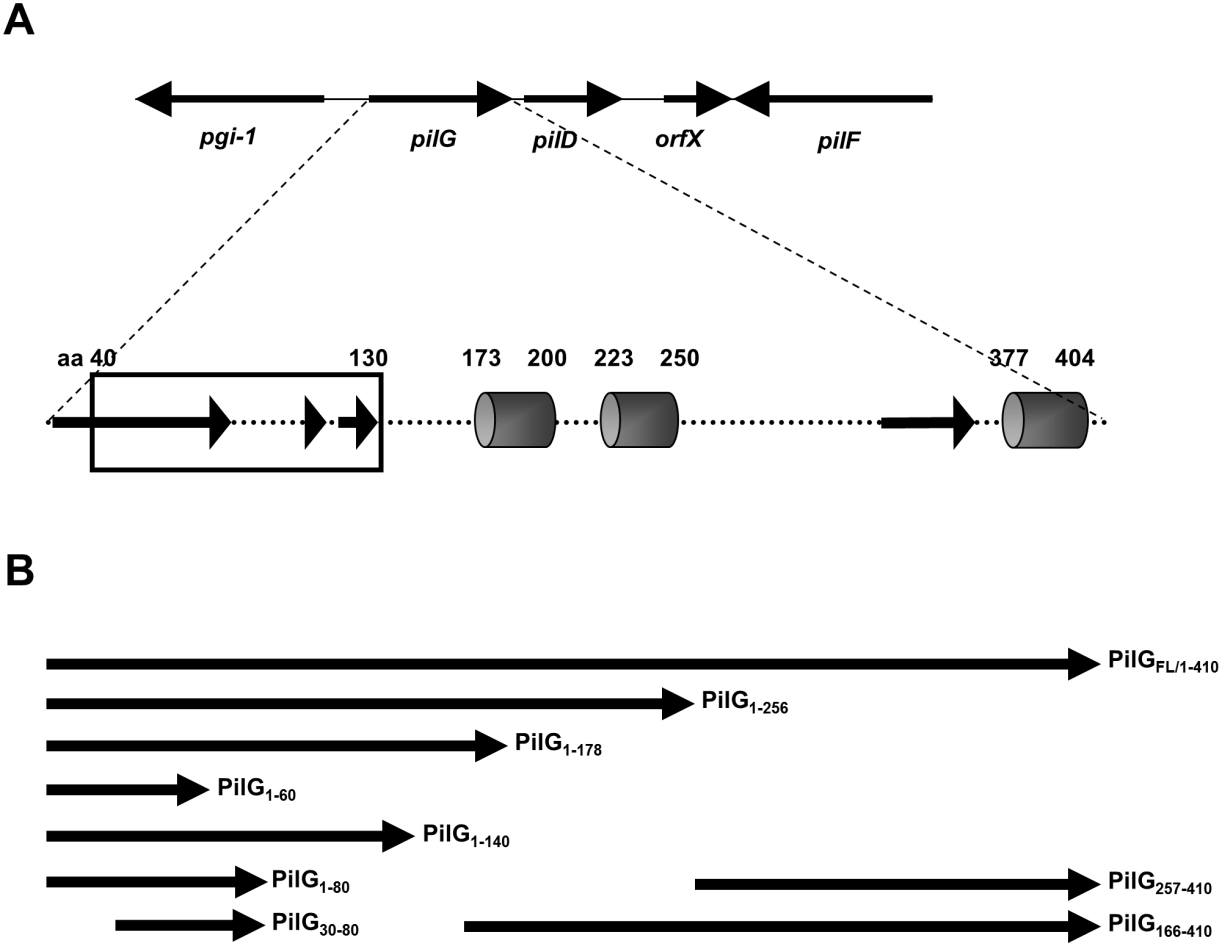
Schematic diagram of *pilG* gene organization. (A) Arrangement of *pilG* and flanking genes in *N*. *meningitidis* MC58 genomic DNA and structural predicted features of the PilG polypeptide. Transmembrane helices are indicated by gray cylinders and disordered regions by black arrows. A predicted DNA binding motif at the N-terminus of PilG is indicated by a black rectangle. (B) Schematic diagram of PilG expression constructs used in this study. All constructs contain a C-terminal 6× His-tag and the name of each construct is indicated to the right.

All DNA manipulations were performed according to standard techniques [52]. Full-length (FL) *pilG* and partial constructs were PCR-amplified from MC58 genomic DNA by using the primers listed in S1 Table. Each fragment was cloned into the vector pET28b(+) (Novagen) with a C-terminal 6X His-tag, yielding a panel of constructs encoding N- and C-terminal parts of *pilG* (see Table 1). The recombinant proteins were overexpressed in *E*. *coli* ER2566 (New England Biolabs). A schematic representation of the PilG constructs used in this study is given in Fig 1B, while all the PilG constructs made are summarized in S1B Fig. The results for the expression of the partial PilG constructs, also showing the one which did not yield expressed proteins, are summarized in S2 Table.

### Site-directed mutagenesis of PilG

PilG was targeted for amino acid substitution with alanine between residues 3 to 63, using the QuickChange Site-Directed Mutagenesis technique (Stratagene). Primers were designed based on the method evaluation carried out by Zheng and co-workers [53], and the vector pPilG1 expressing full-length PilG was used as a template (Table 1 and S1 Table). In the process of the experiments an unintended mutation (E41H) in pPilG-E41H/RKK43-45AAA was discovered and corrected to give the plasmid pPilG-RKK43-45AAA.

### Purification of recombinant PilG proteins

Since the recombinant proteins produced have different solubility (S2 Table), the following optimized methods were used to purify full-length and partial PilG proteins.

#### (I) Membrane associated proteins

*E*. *coli* ER2566 cells harboring plasmids that encoded PilG_FL_, PilG_1-256_, PilG_257-410_, PilG_166-410_, PilG_K3A_, PilG_KK12-13AA_, PilG_E14A_, PilG_KR15-16AA_, PilG_EEE39-41AAA_, PilG_RKK43-45AAA_, PilG_E41H/RKK43-45AAA_, PilG_Q55A_ or PilG_SS62-63AA_ were grown at 37°C in LB medium containing 50 μg ml^-1^ kanamycin. The cultures were cooled to 18°C at OD_600_ = 0.6, after 30 min induced with 0.5 mM isopropyl-D-thiogalactopyranoside (IPTG) and grown at 18°C overnight. The cells were harvested by centrifugation at 4000 × g for 20 min and frozen at -70°C. The cell pellet was resuspended in phosphate buffer (50 mM NaH_2_PO_4_, 300 mM NaCl, pH 8.0) with the protease inhibitor Complete without EDTA (Roche Applied Science) and benzonase (Merck), and lysed by passing three times through a French press (103.500 kPa, Thermo Electron) or by sonication at ≈100 W (80% output, Branson Scientific) 10 × 10 s. Unbroken cells were removed twice by centrifugation for 10 min at 4000 × g and the membrane-enriched fraction was collected by ultracentrifugation (150.000 × g, 90 min). The membrane pellet was resuspended in phosphate buffer (pH 8.0) containing 10 mM imidazole and 1% n-Dodecyl β-D-maltoside (DDM, Glycon) and was left to solubilise on a roller at 4°C overnight. Unsolubilised material was removed by ultracentrifugation (150.000 × g, 90 min). The supernatant was added to a Ni-NTA column (Qiagen), washed and eluted with phosphate buffers (pH 8.0) containing 0.1% DDM and increasing amounts of imidazole up to 250 mM. Fractions containing the recombinant proteins were pooled and dialyzed against phosphate buffer (pH 8.0) containing 0.1% DDM.

#### (II) Soluble partial protein

*E*. *coli* ER2566 cells harboring plasmids encoding PilG_1-60_, PilG_1-80_, PilG_1-140_ and PilG_1-178_ were grown and lysed as previously described. Unbroken cells and cell debris were removed by centrifugation twice at 20.000 × g for 20 min. The supernatant was added to a Ni-NTA column (Qiagen), washed and eluted with phosphate buffers (pH 8.0) containing increasing amounts of imidazole up to 250 mM. Fractions containing the recombinant proteins were pooled and dialyzed against phosphate buffer (pH 8.0).

#### (III) Purification of inclusion bodies

PilG partial protein PilG_30-80_ was purified by isolation of inclusion bodies, solubilization in urea and protein purification with Ni-NTA under denaturing conditions according to protocols provided by Qiagen [54]. Urea was removed by dialysis against a phosphate buffer (pH 8.0), yielding a soluble protein. A complete list of PilG_FL_ and partial recombinant proteins purified is available in S2 Table.

### SDS-PAGE and immunoblotting

Procedures for SDS-PAGE and immunoblotting have previously been described [7, 17]. The samples were mixed with an equal amount of sample buffer (20% glycerol, 5% β-mercaptoethanol, 0.05% bromophenol blue, 125 mM Tris-HCl, pH 6.8) and kept on ice for 15 min before gel electrophoresis in a Mini-PROTEAN system (Bio-Rad) at 4°C, with a constant voltage of 110 V.

### Rabbit immunization and antibody production

Rabbit polyclonal antibodies were raised against the PilG_FL_ protein as previously described [20]. Serum obtained 100 days after immunization was used to detect PilG. The anti-PilQ sera used were previously described [55].

### South-western analysis

The DNA binding ability of PilG_FL_, partial proteins and alanine substitution mutants was assessed by a solid phase DNA overlay assay as previously described [38]. The DNA substrates used in the assay are listed in Table 2. Purified DNA glycosylase Fpg [56] and bovine serum albumin (BSA) were used as positive and negative controls, respectively. Each experiment was repeated at least three times.

**Table 2:** DNA substrates employed in the study.

Substrate	Sequence (5´→3´)^a^	DUS
**ssDNA**		
T_1_	CAACAACAACAACAGCCGTCTGAACCAAATTCAGACGGCAACAACAACAACA	DUS
T_3_	CAACAACAACAACAGGCCTGTCATCCAAAATGACAGGCCAACAACAACAACA	-
HH7^b^	AACAACAACAAATGCCGTCTGAACCAACATGCCGTCTGAAAACAACAACAAC	AT-DUS
HH8	GTTGTTGTTGTTTTCAGACGGCATGTTGGTTCAGACGGCATTTGTTGTTGTT	AT-DUS
HH9	GTTGTTGTTGTTATGCCGTCTGAAGTTGGATGCCGTCTGAATTGTTGTTGTT	AT-DUS
HH10^b^	AACAACAACAAAAGGCCTGTCATCCAACTTCCGGACAGTAAACAACAACAAC	-
**dsDNA**		
T_1_T_2_	CAACAACAACAACAGCCGTCTGAACCAAATTCAGACGGCAACAACAACAACA	DUS
	TGTTGTTGTTGTTGCCGTCTGAATTTGGTTCAGACGGCTGTTGTTGTTGTTG	
T_3_T_4_	CAACAACAACAACAGGCCTGTCATCCAAAATGACAGGCCAACAACAACAACA	-
	TGTTGTTGTTGTTGGCCTGTCATTTTGGATGACAGGCCTGTTGTTGTTGTTG	
HH7HH8 ^b^	AACAACAACAAATGCCGTCTGAACCAACATGCCGTCTGAAAACAACAACAAC	AT-DUS
	GTTGTTGTTGTTTTCAGACGGCATGTTGGTTCAGACGGCATTTGTTGTTGTT	
HH10HH11^b^	AACAACAACAAAAGGCCTGTCATCCAACTTCCGGACAGTAAACAACAACAAC	-
	GTTGTTGTTGTTTACTGTCCGGAAGTTGGATGACAGGCCTTTTGTTGTTGTT	

^a^ DUS is underlined.

^b^ DNA substrates used in EMSA.

### Labeling of DNA substrates

Oligonucleotides were end-labeled with [γ^32^P]ATP (Perkin Elmer) using T4 polynucleotide kinase (New England Biolabs) as described by the manufacturer. Labeled oligonucleotides were separated from free nucleotides on 20% non-denaturing gels by PAGE and extracted by diffusion into water. Double-stranded labeled substrates were generated by mixing labeled oligonucleotides with an equal molar amount of complementary unlabeled oligomer, heating to 95°C for 5 min and slow cooling to room temperature. The concentration of the double-stranded DNA substrate was estimated by dot quantification on agarose plates containing ethidium bromide [52], using unlabeled DNA of known concentration as the standard.

### Electrophoretic mobility shift assay (EMSA)

In the first analysis for determining the half maximal effective concentration (EC_50_) for PilG_FL_ and the PilG alanine substitution constructs, the binding reaction was performed for 15 min on ice in a buffer containing 25 mM NaH_2_PO_4_, 150 mM NaCl, 1.0 mM DTT, 10% [w/v] glycerol, and 0.05% [w/v] DDM at pH 8.0. In later experiments using an optimized EMSA with the PilG partial proteins PilG_1-178_, PilG_1-80_ and PilG_30-80_, 4.5 fmol labeled DNA was mixed with 5 μl 2× gel shift buffer, yielding a final concentration of 50 mM HEPES, 1.0 mM DTT, 100 mM NaCl, 5 mM MgCl_2_, 10% [w/v] glycerol, 0.05% [w/v] DDM, pH 7.5, and protein in a final volume of 10 μl. These mixtures were incubated on ice for 15 min. Electrophoresis was carried out on 4% or 6% polyacrylamide gels in Tris/glycine/EDTA buffer [57]. Gels were dried, exposed in a PhosphorImager cassette, scanned in a Typhoon scanner and quantified using ImageQuant (GE Healthcare).

### Endoproteinase cleavage of N-terminal PilG

In order to map the location of the DNA binding domain of PilG and the site mediating protein-protein interactions, the N-terminal recombinant proteins PilG_1-80_ and PilG_1-178_ were cleaved with endoproteinase Asp-N enzyme (Roche Applied Science). Briefly, 400 μg of purified protein was mixed with 2 μg of lyophilized endoproteinase Asp-N sequencing grade enzyme. 1 M urea and 0.01% SDS were added to the proteolysis reaction in order to resolve protein folding, which might shield enzyme-specific cleavage sites. The reaction was conducted at 37°C and aliquots were collected at selected time points for a period of 24 h. All samples were separated by SDS-PAGE and DNA binding activity of the cleavage products was determined by South-western analysis. The most predominant Asp-N cleavage products were excised from the Coomassie Blue-stained gel, further in-gel digested with trypsin, and the obtained peptides were identified by mass spectrometry (MS) analysis.

### Peptide identification by mass spectrometry

PilG fragments selected from the endoproteinase cleavage assay were identified by peptide mass fingerprinting/MS as previously described [58]. In brief, tryptic peptides obtained from in-gel digestion were desalted and concentrated using C18 3M Empore Extraction Disks (Varian) placed in GELoader tips (Eppendorf). The peptides retained were eluted onto a stainless steel target plate with a solution containing 70% acetonitrile, 0.1% trifluoroacetic acid and 10 mg ml^-1^ α-cyano-4-hydroxycinnamic acid. The samples were analyzed on an Ultraflex II MALDI-TOF/TOF-mass spectrometer (Bruker Daltonics) operated in the positive reflector mode. For peptide identification, MALDI-TOF spectra were compared to the PilG sequence after in silico digestion with Asp-N and trypsin using the software Biotools v3.0.

### Far-western analysis

Protein-protein interactions between PilG_FL_, partial proteins and endoproteinase cleavage products, in addition to other pilus biogenesis proteins, were assessed by a solid phase protein overlay assay as previously described [11, 17]. Briefly, 1 μg of purified recombinant PilG, PilQ [55], PilN, PilO, PilP [11], PilF, PilT, ComP, ComL proteins, purified pili [59] and BSA were applied to SDS-PAGE and transferred onto nitrocellulose membranes (Hybond-C Extra, Amersham Biosciences) in Towbin transfer buffer (25 mM Tris-HCl, 192 mM glycine, 20% methanol, 0.1% SDS, pH 8.3). The membranes were briefly washed twice with renaturing buffer (0.25% gelatin, 0.5% BSA, 0.2% Triton X-100, 10 mM Tris-HCl, 5 mM β-mercaptoethanol, 100 mM NaCl, pH 7.5), and the proteins were renatured by incubation at 4°C overnight in the same buffer. For the detection of protein-protein interaction, the membranes were incubated for 3 h with 1 μg purified full-length PilG or PilQ or with partial proteins thereof in 10 ml renaturing buffer, and washed in a Tris-buffered saline (100 mM Tris-HCl, 150 mM NaCl, pH 7.5). Bound PilG or PilQ were detected with specific rabbit antisera. The PilQ-PilP interaction was used as a positive control [11], while BSA was used as a negative control. All the experiments were repeated at least three times.

### Expression of mutant *pilG in vivo*

To analyze the biological significance of the amino acids involved in DNA binding the mutations *pilG*_*EEE39-41AAA*_ and *pilG*_*RKK43-45AAA*_ were introduced into Mc. For this, the *pilG* was amplified from Mc with the primers SF101 and SF134, the *aph* was amplified form pUP6 with the primers OHA_AphEcoRI_REV and SAF-Tn5-aph-for, and the 5’ *pgi* with the primers SF135 and SF136. The PCR products were digested with the appropriate restriction enzymes (S2 Table) and as a concatenate ligated into pBluescript II SK(+) (Stratagene) giving the vector pSAF67. The mutations to generate *pilG*_*EEE39-41AAA*_ and *pilG*_*RKK43-45AAA*_ were introduced into pSAF67 giving the vectors pSAF68B and pSAF69, respectively (Table 1). The wild-type and mutant *pilG* genes were transformed into the Mc strain MC58. Positive clones were selected for by kanamycin resistance and the mutations in *pilG* confirmed by DNA sequencing and mass spectrometry of the expressed proteins. Pilus expression and PilG expression were confirmed by immuno blot (S10 Fig). The mutant strains were tested by quantitative competence screening as described below.

### Phenotypic analysis of *N*. *meningitidis* PilG site-directed mutants

*N*. *meningitidis* site-directed mutants were compared to wild type strains in phenotypic analysis.

#### (I) Colony morphology

*N*. *meningitidis* strains cultured on clear GC plates were assessed by stereo microscopy to define whether they had a piliated (P+) or non-piliated (P-) colonial morphology [60].

#### (II) Purification of type IV pilus fibers

Type IV pili were purified following the short method as described by Brinton [61]. The pilus preparations were separated by SDS-PAGE, blotted onto nitrocellulose membrane and immunoblot was performed with a pilin specific antiserum.

#### (III) Competence screening

Competence for the transformation of wild type and mutant strains was performed using the plasmid pOHA-D4 as donor DNA (Table 1). Wild type and mutant strains were harvested in a CO_2_-saturated liquid GC medium containing 7 mM MgCl_2_ and 1× IsoVitaleX. The bacteria were exposed to either plasmid pOHA-D4 or distilled water (negative control) for 45 min before adding 10 volumes of liquid GC medium. The bacterial solutions were incubated with tumbling at 37°C for 4.5 h and subsequently diluted and plated on both plain GC medium and GC medium containing erythromycin (Erm) [8 μg ml^-1^]. The transformation rate was calculated by dividing the number of Erm-resistant colony forming units (c.f.u.) by the total number of c.f.u. The assay was repeated at least three times for each null mutant.

### Separation of outer and inner membranes by sucrose density gradient

Mc outer and inner membranes were separated by sucrose density gradient as previously described [11]. In brief, Mc M1080 cells were washed with phosphate buffered saline (PBS), resuspended in 50 mM Tris buffer, pH 8.0, containing 50 μg RNase and DNase (Sigma) and processed twice through a French press (103.500 kPa, Thermo Electron). The debris was removed by centrifugation at 10.000 × g for 10 min. Sucrose gradient centrifugation was carried out in water with 3 mM EDTA, pH 8.0 [62]. The sample was transferred onto two layers of sucrose consisting of 3 ml of 55% [w/v] and 4 ml 15% sucrose, and centrifuged at 217.000 × g and 4°C for 5 h in a SW40Ti rotor (Beckman). The membrane fraction positioned at the interface was collected and diluted down to 30% sucrose, applied to a discontinuous sucrose gradient consisting of 3 ml of 50, 45, 40 and 35% sucrose, and centrifuged in a SW40Ti rotor at 180.000 × g and 4°C for 35 h. After fractionation, 10 μl samples were analyzed by SDS-PAGE, followed by Coomassie Blue-staining or immunoblotting.

## Results

### PilG is predicted to have a putative DNA binding domain

Alignments of the predicted amino acid (aa) sequences of PilG and orthologs revealed sequence conservation around residues 70 to 100, 120 to 220 and 270 to 400, while there is only little conservation at residues 1 to 60 (S1 Fig). To search for DNA binding motifs, we focused on three regions of the PilG aa sequence, residues 1 to 185, 200 to 225 and 250 to 377, and excluded predicted transmembrane helices between these segments. This in silico analysis suggested that a putative PilG DNA binding domain localizes to residues 40 to 130 (Fig 1A). Bioinformatics analysis of the PilG aa sequence showed no other commonly recognized/conserved functional domains or signature motifs.

### Estimating the DNA binding capacity of PilG

Our previous study showed that full-length PilG (PilG_FL_) binds single-stranded DNA (ssDNA) and double-stranded DNA (dsDNA) without DUS specificity [38]. In this study, we further extended the analysis to quantify the DNA binding activity of PilG at low DNA concentration (0.45 nM) and high PilG concentration (≥94 nM) using an EMSA. These conditions were selected as the protein concentration required to bind half of the input DNA approximates the dissociation constant, K_d_, of the protein-DNA complex [63]. In this preliminary EMSA assay using a 52 base pair (bp) dsDNA substrate, the estimated apparent PilG K_d_ value for PilG_FL_ was 0.3 μM (S3 Fig).

### N-terminal PilG binds DNA

**Fig 2:**
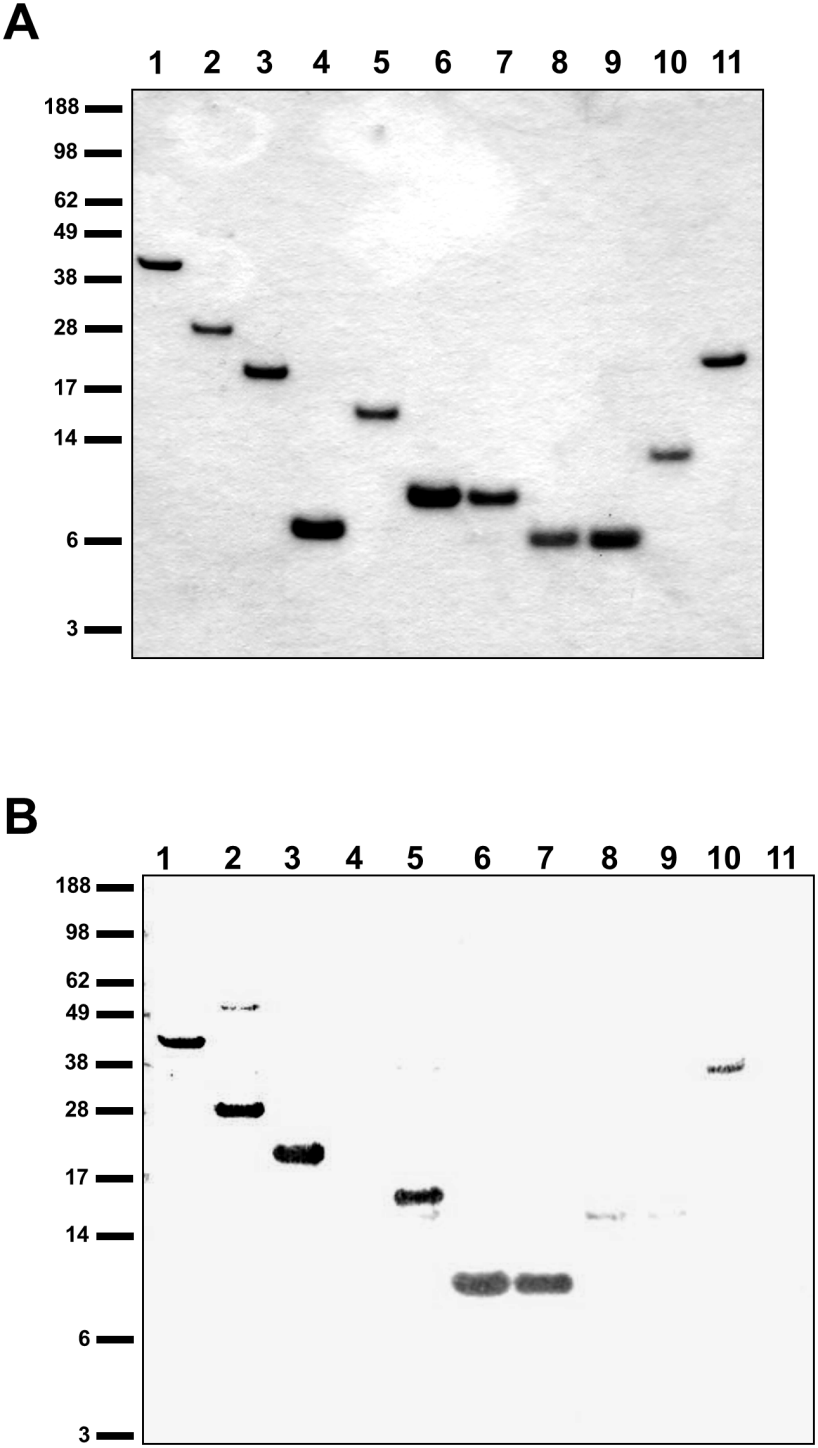
DNA binding activity of PilG full-length and partial recombinant proteins. (A) Coomassie Blue-staining of recombinant proteins separated by SDS-PAGE. Lane 1, PilG_FL/1-410_; lane 2, PilG_1-256_; lane 3, PilG_1-178_; lane 4, PilG_1-60_; lane 5, PilG_1-140_; lane 6, PilG_1-80_; lane 7, PilG_1-81_; lane 8, PilG_30-80_; lane 9, PilG_30-81_; lane 10, PilG_257-410_; lane 11, PilG_166-410_. (B) Solid phase overlay assay of the proteins shown in A. Similar results were obtained with all DNA substrates employed (see Table 2). Additional bands in lane 2, 8 and 10 constitute *E*. *coli* contaminants due to differences in purity between the recombinant proteins. The positions of the protein size standards (kDa) are shown on the left.

**Fig 3:**
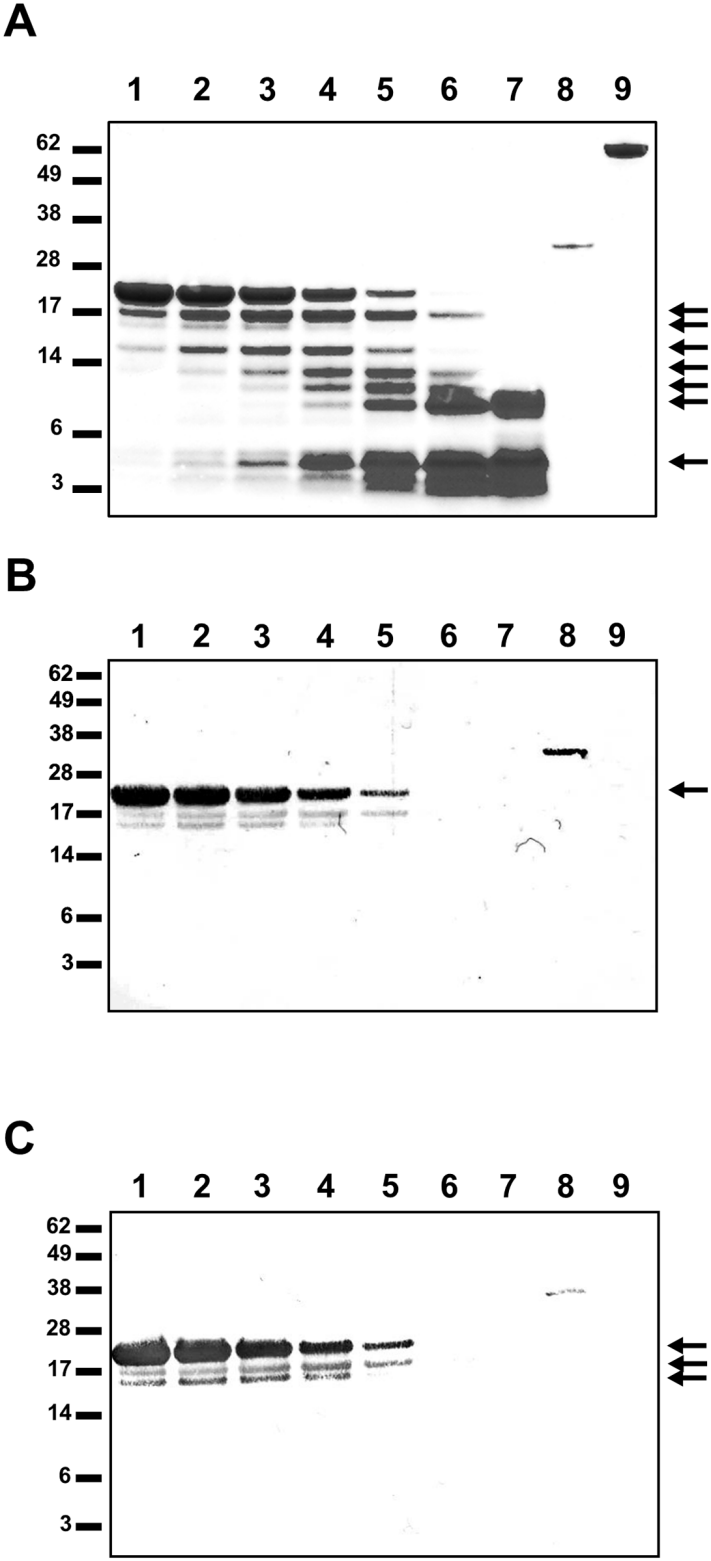
Endoproteinase cleavage of PilG_1-178_. (A) Endoproteinase-cleaved PilG_1-178_ analyzed by SDS-PAGE and protein staining with Coomassie Blue. South-western assay of the cleavage products with DUS containing (B) ssDNA or (C) dsDNA. Protease incubation times are for lane 1, 15 min; lane 2, 30 min; lane 3, 1 h; lane 4, 2 h; lane 5, 4 h; lane 6, 8 h; lane 7, 24 h. Lane 8 contains Fpg and lane 9 contains BSA. The positions of the protein size standards (kDa) are shown on the left and the arrows indicate protein bands analyzed by mass spectrometry. The arrow in B indicates the full-length protein. The three arrows in C indicate the full length PilG_1-178_ and the fragments covering amino acid 1–154 and 27–178.

Based on the bioinformatics analyses, constructs for the expression of truncated PilG proteins were made (Fig 1B and S2B Fig). The truncated variants of recombinant PilG (Fig 1B) were also tested for DNA binding activity using South-western analysis (Fig 2 and Table 3). This analysis mapped the DNA binding activity of PilG to the N-terminal 80 amino acids. In particular, PilG_1-80_ strongly bound DNA (Fig 2, lane 6) while the even shorter partial PilG proteins PilG_1-60_ and PilG_30-80_ did not bind DNA (Fig 2, lanes 4 and 8, respectively). The South-western analysis also yielded no difference in PilG affinity for ssDNA and dsDNA binding, and there was no difference in affinity for DNA with or without the DUS (data not shown), which corresponds to our previous findings for PilG_FL_ [38]. In order to define in more detail which PilG residues contribute to DNA binding, PilG_1-178_ was cleaved with endoproteinase and the cleavage products were evaluated by South-western analysis and mass spectrometry (MS). The cleavage products with DNA binding activity obtained corresponded to residues 1 to 154 and 27 to 178 (Fig 3). When PilG_1-80_ was subjected to endoproteinase cleavage and South-western analysis, residues 1 to 70 was the smallest truncated form of PilG with DNA binding activity (S5 Fig). EMSA assays with dsDNA confirmed that PilG_1-178_ and PilG_1-80_ bound DNA with an affinity similar to that of PilG_FL_, whereas no DNA binding was seen with PilG_30-80_ at the same protein concentration (Table 4). Based on these findings, further investigations mainly focused on the PilG amino acids 1–80, which were required and sufficient for the DNA binding activity.

**Table 3:** Summary of the results of the oligo nucleotide binding tests using recombinant PilG proteins. The “+” and “-”refer to the DNA binding activity of the proteins when analyzed by South-western.

DNA substrates ^a^							
Protein	T_1_	T_1_T_2_	T_3_	T_3_T_4_	HH8	HH7HH8	HH9
PilG_FL/1-410_	+	+	+	+	+	+ ^b^	+
PilG_1-256_	+	+	+	+	+	+	+
PilG_1-178_	+	+	+	+	+	+ ^b^	+
PilG_1-140_	+	+	+	+	+	+	+
PilG_1-80_	+	+	+	+	+	+ ^b^	+
PilG_1-60_	-	-	-	-	-	-	-
PilG_30-80_	-	-	-	-	-	- ^b^	-
PilG_257-410_	-	-	-	-	-	-	-
PilG_166-410_	-	-	-	-	-	-	-
PilG_K3A_	+	nd^c^	nd	nd	nd	+	+
PilG_KK12-13AA_	+	nd	nd	nd	nd	+	+
PilG_E14A_	+	nd	nd	nd	nd	+	+
PilG_KR15-16AA_	+	nd	nd	nd	nd	+	+
PilG_EEE39-41AAA_	+	nd	nd	nd	+	+^b^	+
PilG_RKK43-45AAA_	-^d^	nd	nd	nd	-^d^	-^b^ ^,^ ^d^	-^d^
PilG_E41H/RKK43-45AAA_	-^d^	nd	nd	nd	nd	-^b^ ^,^ ^d^	-^d^
PilG_Q55A_	+	nd	nd	nd	nd	+	+
PilG_SS62-63AA_	+	nd	nd	nd	nd	+	+

^a^ see Table 2.

^b^ confirmed by EMSA.

^c^ not determined.

^d^ reduced binding, see text.

**Table 4:** DNA binding abilities of PilG partial proteins in EMSA. The protein concentration required to achieve a shift with 50% of the DNA substrate is indicated and can be used to estimate the dissociation constant, K_d_, of the protein-DNA complex [63].

PilG protein	DNA substrate	Protein concentrations showing 50% shift of the substrate [μM]
**PilG_1-178_**	dsDNA DUS-	0.19–0.38
**PilG_1-178_**	dsDNA DUS +	0.19–0.38
**PilG_1-80_**	dsDNA DUS +	0.20–0.40
**PilG_30-80_**	dsDNA DUS +	(no shift at up to 1.2 μM)

**Fig 4:**
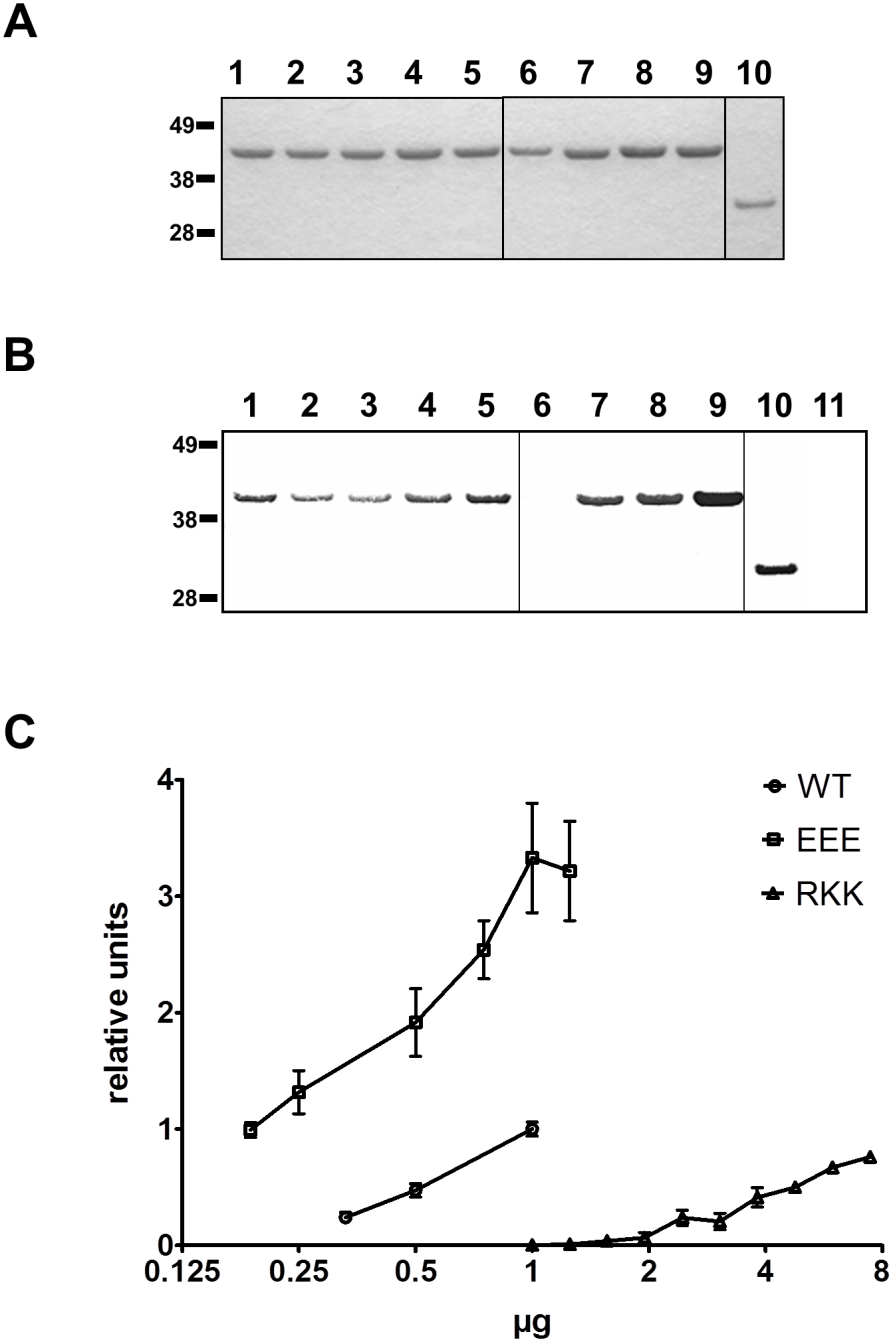
DNA binding activity of PilG alanine substitution mutants. (A) Coomassie Blue-stained SDS-PAGE and (B) South-western assay with the indicated PilG alanine substitution mutants using oligomer HH9 as DNA probe (see Table 2). Protein amount were 1μg/lane for PilG proteins and 0.25μg/lane for Fpg. Lane 1, PilG_FL/1-410_; lane 2, PilG_K3A_; lane 3, PilG_KK12-13AA_; lane 4, PilG_KR15-16AA_; lane 5, PilG_E14A_; lane 6, PilG_E41H/RKK43-45AAA_; lane 7, PilG_SS62-63AA_; lane 8, PilG_Q55A_; lane 9, PilG_EEE39-41AAA_; lane 10, Fpg; lane 11, BSA. Similar results were obtained with all DNA substrates employed (see Table 3). The positions of the protein size standards (kDa) are shown on the left. (C) Relative level of bound DNA substrate South-western at different protein concentrations of recombinant PilG FL (WT), PilG_RKK43-45AAA_ (RKK), and PilG_EEE39-41AAA_ (EEE) measured by densitometry and plotted as relative units with the average value for 1μg PilG FL set to 1. The results of at least three experiments using ssDNA and dsDNA are shown. No significant substrate specificity was detected.

**Fig 5:**
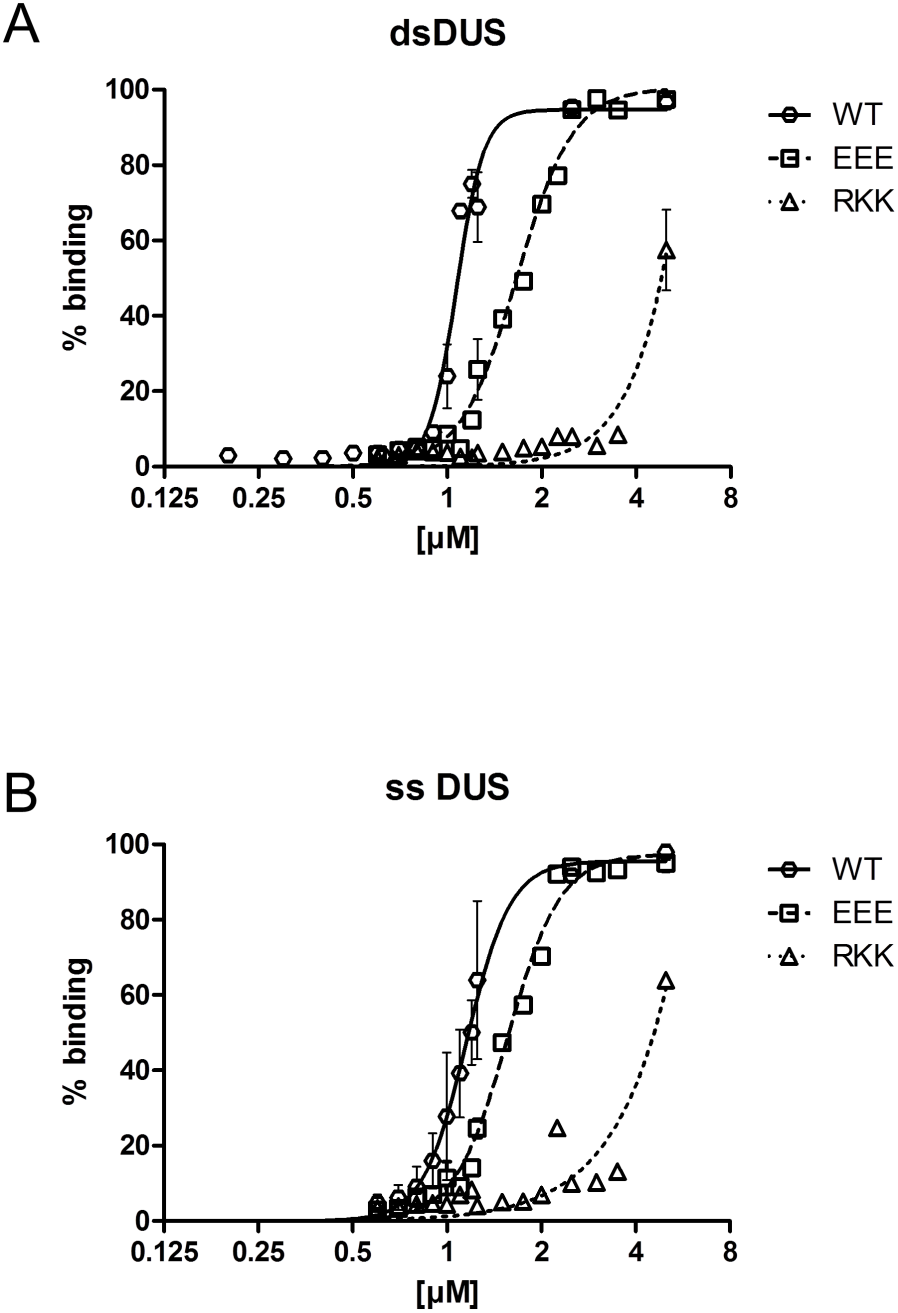
Defining the affinity of PilG to DNA. Plots showing the relative level of bound DNA substrate in EMSA at different protein concentrations of recombinant PilG FL (WT), PilG_RKK43-45AAA_ (RKK), and PilG_EEE39-41AAA_ (EEE) for (A) dsDNA and (B) ssDNA substrate. Shown are the cumulative data from (A) eight and (B) six gel runs and the non-linear fitting curves.

DNA binding sites tend to have positive electrostatic charge and that charged and polar amino acids within DNA binding sites often play a direct role in protein-DNA interactions [64, 65]. Therefore, we expected that alanine substitutions of lysine, arginine, serine and glutamine residues in the N-terminal 80 residues domain of PilG might reduce or abolish DNA binding. Alanine substitutions were introduced into the full-length PilG protein at selected sites (Table 1), including a motif with three consecutive glutamate residues (EEE39-41) and a motif with three consecutive basic residues (RKK43-45) (S1 and S6 Figs). The mutant PilG variants were tested for DNA binding affinity in South-western and EMSA assays (Fig 4 and Table 3). Two of the eight PilG mutants exhibited changes in DNA binding when measured by South-western analysis (Fig 4B). The substitution RKK43-45AAA (with and without the additional substitution E41H) decreased DNA binding affinity (Fig 4B, lane 6, and S7 Fig), while alanine substitution of the glutamic acid residues 39 to 41 increased DNA binding affinity (Fig 4B lane 9). South-western assays using increasing amounts of protein demonstrated that the protein PilG_EEE39-41AAA_ bound DNA with significantly higher affinity than PilG_FL_, while PilG_RKK43-45AAA_ bound DNA with a significantly lower affinity than PilG_FL_ (Fig 4C). The DNA binding affinity of PilG_K3A_, PilG_KK12-13AA_, PilG_E14A_ and PilG_KR15-16AA_ was slightly reduced, while the PilG_Q55A_ and PilG_SS62-63AA_ affinity was the same as of PilG_FL_ (data not shown). To further quantify the DNA binding, the EMSA was optimized (see Methods and compare S3 Fig and S8 Fig). In this shift assay, the PilG_EEE39-41AAA_ and PilG_RKK43-45AAA_, which demonstrated significant differences to wild type PilG_FL_ in South-western analysis, showed slightly different results (Fig 5). Both mutant proteins showed reduced binding to dsDNA as well as ssDNA compared to the wild type protein PilG_FL_. Notably, while the PilG_EEE39-41AAA_ required only a 50% increased value in the protein concentration (yealding half activity, EC_50_), the PilG_RKK43-45AAA_ protein was calculated to need a several hundred-fold increase in protein concentration to reach 50% binding (Table 5), although at the highest protein concentration used (5μM) about 50% binding was seen (Fig 5).

**Table 5:** Best-fit values for k_half_ (= EC_50_, in [μM]) for the EMSA analysis of wild type and mutant PilG proteins using DUS containing substrates. The k_prime_ and h values were calculated using GraphPad Prism 5 [75] and converted into k_half_ values. The goodness of fit is given as R^2^ values in parentheses. Due to the conditions used in the EMSA k_half_ is approximately equal to kd [63].

Protein	dsDNA (HH7HH8)	ssDNA (HH8)
**PilG_FL/1-410_**	1.07 (0.96)	1.16 (0.91)
**PilG_EEE39-41AAA_**	1.67 (0.99)	1.56 (0.99)
**PilG_RKK43-45AAA_**	~6.85×10^2^ (0.90)	~1.57 × 10^4^ (0.89)

**Fig 6:**
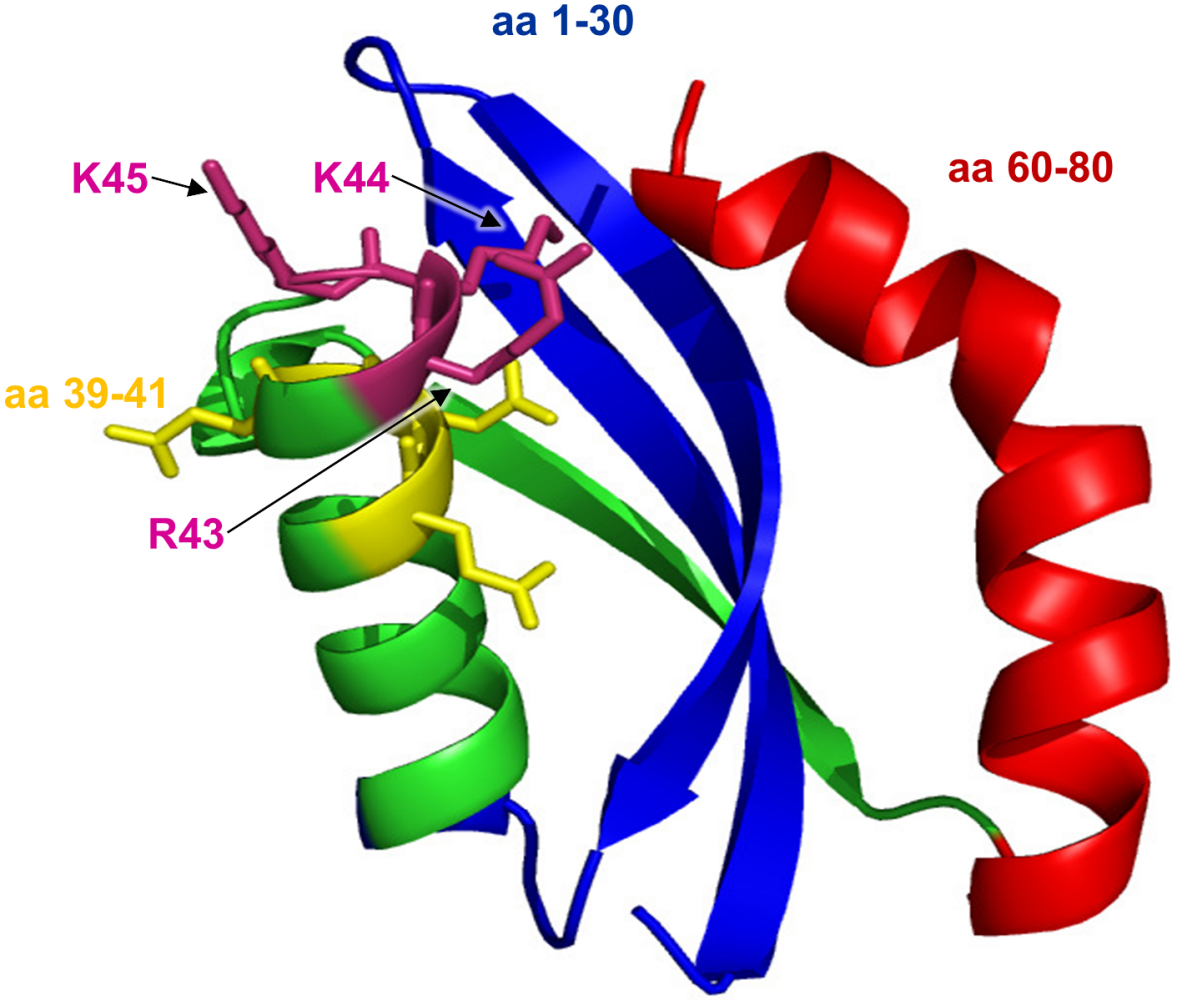
Predicted 3D structure of *N*. *meningitidis* PilG residues 1 to 80. The *N*. *meningitidis* MC58 PilG hypothetical structure generated by the SAM-T06 modeling server is shown in cartoon form with the residues 1 to 30 colored blue and 60 to 80 colored in red, illustrating that these two regions in the folded form are predicted to be closely located. The lysine and arginine residues 43 to 45, which are conserved in Neisseria and showed a role in DNA binding, possibly due to their positive charge, are labelled. In addition, the position of the PilG alanine substitution of mutant PilG_EEE39-41AAA_ is indicated in yellow.

Based on this data, neisserial PilG residues 1 to 30, the RKK motif at aa 43 to 45, and the residues 60 to 80 were predicted to play critical roles in binding of DNA. The results are in line with the structural modeling predicting that these regions are located close to each other in the native PilG (Fig 6).

### PilG amino acids 39–41 and 43–45 affect transformation *in vivo*

**Fig 7:**
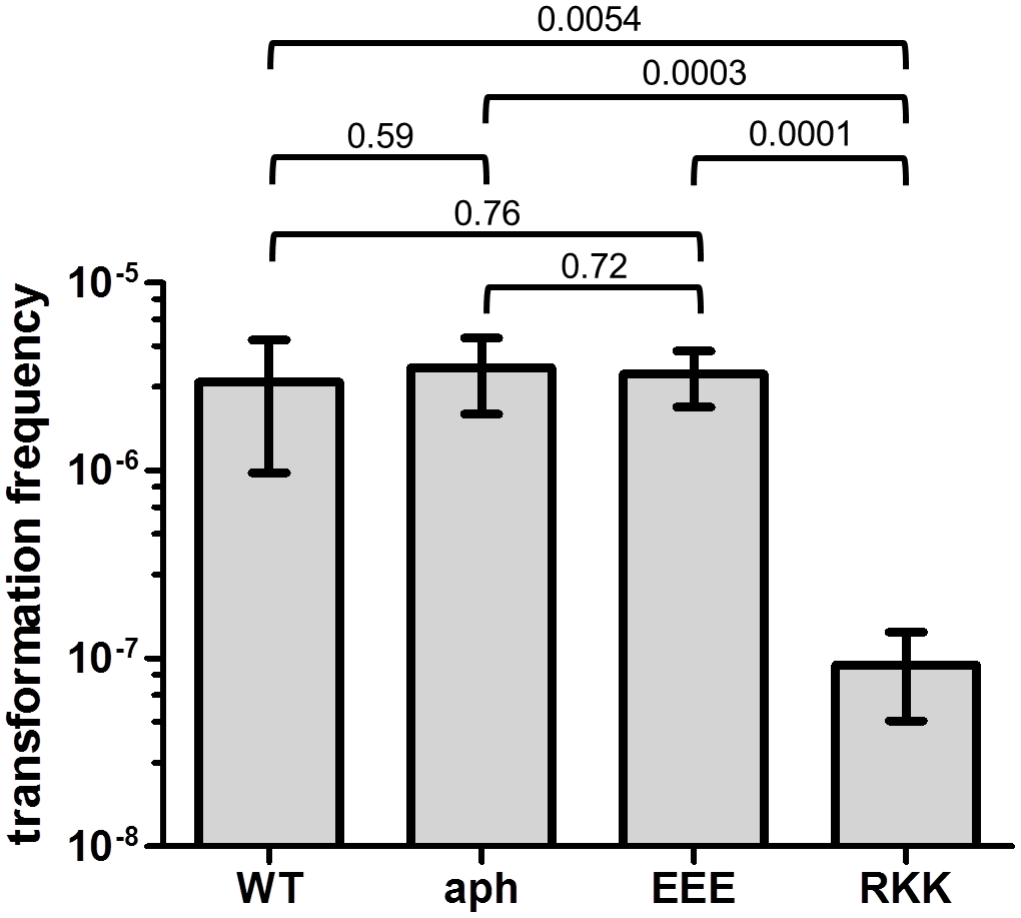
Transformation of *Neisseria meningitidis* expressing PilG variants with altered DNA binding activity. Strain MC58 (WT), MC58-pilG:aph (aph), MC58-pilG-EEE:aph (EEE), and MC58-pilG-RKK:aph (RKK) were used. The strains were quantitatively tested for competence in DNA transformation in six independent experiments. The p values determined by t-test are indicated on the brackets.

To test the significance of the PilG DNA binding in transformation, two alanine substitutions were introduced *in situ* into Mc host cells. Interestingly, DNA transforming activity was significantly reduced in Mc expressing PilG_RKK43-45AAA_ when compared to the wild type (32 times) and the control (38 times) carrying only the selective marker (Fig 7). The efficiency of transformation was however not significantly reduced with the mutation PilG_EEE39-41AAA_ (Fig 7). These *in vivo* findings corroborate the results obtained *in vitro* by EMSA showing the strong negative effect of the PilG_RKK43-45AAA_ mutation on DNA binding.

### N-terminal PilG interacts with N-terminal PilQ

**Fig 8:**
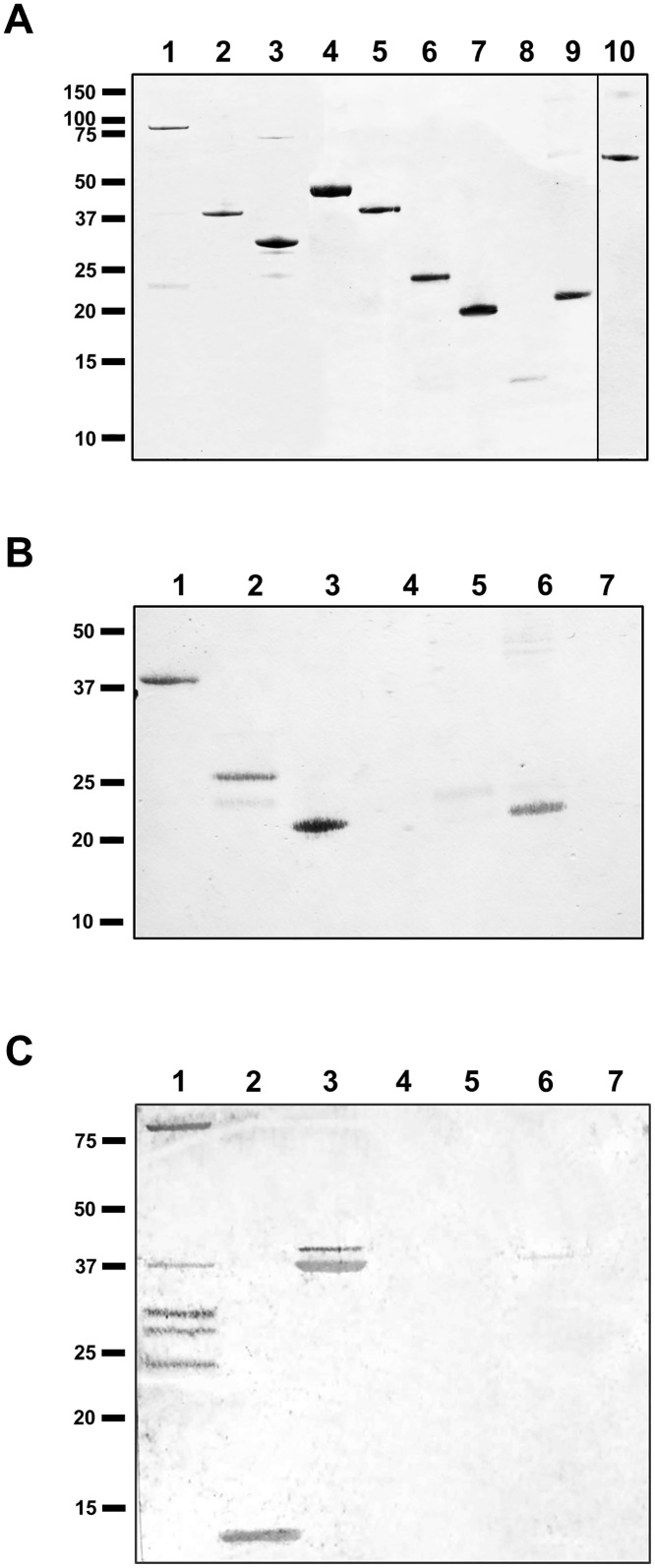
N-terminal PilG directly interacts with N-terminal PilQ monomer. Protein-protein interaction between recombinant meningococcal PilG and recombinant PilQ proteins were detected by a solid phase overlay assay (Far-western analysis). (A) A Coomassie Blue-stained gel showing recombinant proteins used. Lane 1, PilQ_FL/1-761_; lane 2, PilQ_25-354_; lane 3 PilQ_217-478_; lane 4, PilQ_350-761_; lane 5, PilG_FL/1-410_; lane 6, PilG_1-256_; lane 7, PilG_1-178_; lane 8, PilG_257-410_; lane 9, PilG_166-410_; lane 10 (added for comparison only), BSA. (B) Equal amounts of full-length and partial recombinant PilG proteins on the membrane probed with PilQ_25-354_ and then detected with PilQ antiserum. Lane 1, PilG_FL/1-410_; lane 2, PilG_1-256_; lane 3, PilG_1-178_; lane 4, PilG_257-410_; lane 5, PilG_166-410_; lane 6, PilP; lane 7, BSA. (C) Full-length and partial recombinant PilQ proteins on the membrane probed with PilG_1-178_ and then detected with PilG antiserum. Lane 1, PilQ_FL/1-761_; lane 2, PilQ_25-132_; lane 3, PilQ_25-354_; lane 4, PilQ_217-478_; lane 5, PilQ_350-761_; lane 6, PilP; lane 7, BSA. The positions of the protein size standards (kDa) are shown on the left.

Once the localization of PilG in the inner membrane [38] was confirmed (S9 Fig) a solid phase overlay Far-western analysis was employed to assess the interaction between PilG and other pilus biogenesis proteins. Interaction between PilG and PilQ was detected but there was no interaction with PilN, PilO, PilP, PilF, PilT, ComP, ComL or the pilus itself. PilG_FL_, PilG_1-256_ and PilG_1-178_ interacted with PilQ_FL_ and PilQ fragments with PilQ_25-132_ being the shortest fragment showing PilG binding (Table 6 and Fig 8). This suggests that PilG residues 1 to 178 mediate the interaction with N-terminal PilQ. Far-western analysis was also carried out on endoproteinase cleavage products of PilG_1-178_, using PilQ_25-354_ as a probe. MS analysis identified interacting peptides as PilG_27-178_ and PilG_1-154_, further refining the interaction with PilQ to amino acids 27 to 154 (data not shown). We did not detect a direct interaction between PilG and PilT proteins by Far-western analysis (Table 6), although a previous study suggests a functional relationship between neisserial PilG and PilT [4].

**Table 6:** Summary of Far-western analysis assessing the interaction between PilG and other pilus biogenesis proteins. The “+” refers to positive and the “-”refers to no protein-protein interaction detected.

	PilG_FL/1-410_	PilG_1-256_	PilG_1-178_	PilG_257-410_	PilG_166-410_
PilQ_FL/1-761_	+ ^a^	+ ^a^	+ ^a^	-	-
PilQ_25-132_	+ ^a^	+ ^a^	++ ^a^	-	-
PilQ_25-354_	+ ^a^	+ ^a^	+ ^a^	-	-
PilQ_217-478_	-	-	-	-	-
PilQ_350-761_	-	-	-	-	-
PilN	-	-	-	-	-
PilO	-	-	-	-	-
PilP	-	-	-	-	-
PilF	-	-	-	-	-
PilT	-	-	-	-	-
ComP	-	-	-	-	-
ComL	-	-	-	-	-
Pilus	-	-	-	-	-
BSA	-	-	-	-	-

^a^ results confirmed by testing both components alternating in liquid and bound to solid phase.

## Discussion

DNA uptake through natural transformation is a dynamic process that mediates allelic replacement of genes into the genome in many bacteria [66, 67]. Transformation is an important process influencing bacterial fitness and survival, and may also increase the spread of antibiotic resistance among bacterial species [68]. In neisserial species, transformation is dependent on type IV pili. Hence, in order to better understand the mechanism by which DNA is taken into neisserial cells, we studied the DNA binding and protein interaction properties of the pilus biogenesis protein PilG.

The fact that PilG null mutants are non-piliated emphasizes the role of PilG in pilus biogenesis [20]. We have previously shown that PilG co-purifies with the inner membrane, suggesting that it is in direct contact with both the cytosol and the periplasm. As indicated by Derrick and co-workers in their PilG structural analysis [19], N-terminal PilG is predicted to be oriented into the cytoplasm. Here, we demonstrate that the N-terminal region of PilG binds DNA in a DUS independent manner, indicating that PilG is not a selective factor in the DNA transport in *Neisseria sp*. In addition, N-terminal PilG directly interacts with N-terminal PilQ.

Our results demonstrated that PilG N-terminal residues 1 to 30 and 60 to 80 were required for DNA binding activity. Because the isoelectric points of PilG_1-80_ and PilG_30-80_ are 10.58 and 10.59, respectively, DNA binding by PilG_1-80_ cannot be explained solely by overall electrostatic charge. By analyzing PilG alanine substitution mutants *in vitro*, we determined that PilG residues EEE39-41 and RKK43-45 exert negatively and positively modulating effects on PilG DNA binding, respectively. Structural modeling studies predicted that the residues 1 to 30 and 60 to 80 are located close to each other in native PilG (Fig 6). We propose that these two PilG regions cooperatively form a DNA binding site.

PilG DNA binding affinity, as monitored by EMSA (Fig 5 and Table 5), was rather low (K_half_ ≈ 1.16 μM for ssDNA and K_half_ ≈ 1.07 μM for dsDNA). Nonetheless, the competence protein ComEA of *Bacillus subtilis* has similarly low affinity for a 112 bp dsDNA substrate (K_d_ ≈ 0.5 μM) [69]; thus, the PilG-mediated DNA binding activity is comparable to that of other validated DNA binding proteins involved in DNA transformation. Importantly, we observed that PilG_EEE39-41AAA_ has only a slightly reduced DNA binding activity (Table 5) and its expression has no significant negative effect on DNA transformation, while PilG_RKK43-45AAA_ has a strongly reduced DNA binding activity and *in vivo* significantly reduces DNA transformation (Fig 7). This result shows that the DNA binding activity of PilG plays a biologically significant role, facilitating DNA uptake during transformation of neisserial species.

While PilG residues RKK43-45, together with residues 1 to 30 and 60 to 80, appear to be of prime importance for DNA binding, the more C-terminally located part of N-terminal PilG may contribute to protein-protein interactions that are also important during DNA uptake and transformation. As documented by our combined endoproteinase cleavage and Far-western analysis, as well as Far-western analysis with partial PilG proteins, PilG residues 80 to 154 contribute significantly to the interaction with N-terminal PilQ. We propose that highly conserved lysine and arginine residues in PilG_1-30_ and PilG_60-80_, forming a positively-charged protein region, may facilitate both protein-DNA and protein-protein interactions. Positive electrostatic surfaces are commonly found in DNA binding sites and can also promote binding to negatively-charged membranes, receptors or other proteins [70]. However, differences between protein-DNA and protein-protein interaction sites in terms of polarity and charge have been described [46].

The observation that N-terminal PilG binds to N-terminal PilQ underscores the importance of PilG in pilus biogenesis and could be mediated by the short predicted periplasmic loop of PilG [19]. Previous studies have shown that the 900 kDa PilQ complex spans both the outer and inner membranes [11], undergoes structural conformation changes upon binding to type IV pili [17] and exhibits DNA binding activity [10]. In addition, the finding that N-terminal PilQ directly interacts with PilG is consistent with previous findings that N-terminal PilQ binds DNA [10] and is directed towards the inner membrane [55]. Structural modeling of these interactions was previously reported [27].

Exogenous DNA is thought to pass through the PilQ pore during DNA uptake [10]. Subsequently, PilG could guide the DNA into the cytoplasm, acting as an intermediate chaperone in the vicinity of the inner membrane. Since N-terminal PilQ also binds DNA [10], the PilG DNA binding site could potentially interact with PilQ and transforming DNA in a coordinated or sequential way. This interaction of the cytoplasmic N-terminal part of PilG could be allowed by the linking of the cytoplasm and periplasm through the PilG multimer platform structure which was suggested earlier [19]. It is also possible that PilG and the ComA pore might cooperatively facilitate DNA uptake. The observation that *B*. *subtilis* RecA co-localizes with the competence machinery indicates that DNA uptake and recombination are closely linked in space and time [71]. Therefore, PilG could potentially play direct roles in both competence and DNA recombination.

Taken together, our data suggest that the N-terminal domain of PilG is a DNA binding site that potentially operates in conjunction with N-terminal PilQ. These findings point to the biological significance of PilG/PilQ-mediated DNA binding and more comprehensive functional and structural studies of PilG are needed. These should address questions about PilG multimerization, topology, interactions with pilus biogenesis proteins and DNA, and, in particular, more precise identification of the residues and motifs that enable these specific interactions. More complete elucidation of the biological function(s) of PilG will likely improve our understanding of neisserial transformation and hence antigenic variation and antibiotic resistance.

## Supporting Information

S1 Fig Alignment of PilG orthologs from naturally competent bacterial species. The full amino acid sequences of PilG orthologs from neisserial species and other competent bacteria were aligned using Clustal. Amino acids for alanine substitution-mutation, which were made in the progress of this study, are underlined and numbered.
(TIF)

S2 Fig Schematic presentation of the locations of all PilG constructs made in this study. (A) Structural features as presented in Fig 1A. (B) All PilG constructs in position related to A with the names indicated to the right. All constructs contain an additional C-terminal 6×His-tag.
(TIF)

S3 Fig Preliminary test of the DNA binding activity of PilG_FL_ protein. EMSA was performed on PilG_FL_ recombinant protein incubated with a [γ^32^P]ATP labeled 52 bp dsDNA DUS containing substrate (HH7HH8) in the phosphate buffer system described in the methods part. For the controls see S4 Fig Based on three experiments the estimated concentrations required for half-maximal binding activity was 0.3 μM. Protein concentrations are given on top of the lanes in [μM]. The positions for the free DNA and the DNA-PilG_FL_ complex are indicated by arrows.
(TIF)

S4 Fig EMSA controls. EMSA was performed with (A) the positive control protein Fpg and (B) with BSA as negative control. On top the lanes the concentrations of protein used are indicated, [nM] for Fpg and [μM] for BSA. The radiolabeled DNA used was double-stranded and without DUS. Positions for the free DNA and the DNA-Fpg complex are indicated by arrows.
(TIF)

S5 Fig Endoproteinase cleavage of PilG_1-80_. (A) Coomassie Blue-stained gel of endoproteinase cleaved PilG_1-80_. The DNA binding activity of the cleavage products was assessed with a solid phase overlay assay using 10 bp DUS^+^ (B) ssDNA and (C) dsDNA as substrates. Lanes 1 to 7 contain samples after different digestion times (1, 15 min; 2, 30 min; 3, 1 h; 4, 2 h; 5, 4 h; 6, 8 h; 7, 24 h, respectively) and lane 8 contains the positive control, Fpg. The positions of the molecular size markers are shown in kDa on the left. The arrow indicates the full-length protein.
(TIF)

S6 Fig Electrostatic potential distribution of the N-terminal part of PilG. The (B) electrostatic charge on the protein surface of (A) the predicted structure for the N-terminal PilG (aa 1–80) was computed using PyMol. (B) The negative charged area (coloured in red) due to the amino acids 39–41 EEE and the positive charged area (coloured in blue) due to the amino acids 43–45 RKK are encircled.
(TIF)

S7 Fig DNA binding activity of PilG alanine substitution mutants. (A) Coomassie Blue-staining confirms equal amounts of protein loaded for the assay shown in panel B. (B) A solid phase overlay assay for protein-DNA interaction shows the DNA binding activity of PilG_FL_ compared to the PilG alanine substitution mutants PilG_RKK43-45AAA_, PilG_E41H/RKK43-45AAA_ and PilG_EEE39-41AAA_. The DNA substrate used was double-stranded containing AT-DUS. Fpg and BSA were used as positive and negative controls, respectively. PilG_RKK43-45AAA_ and PilG_E41H/RKK43-45AAA_ showed reduced DNA binding compared to PilG_FL_ and PilG_EEE39-41AAA_. The positions of the molecular size markers are shown in kDa on the left. Protein amounts are given on top of the lanes. The arrow indicates an unknown contaminant that is not visible by protein staining in the PilG_RKK43-45AAA_ sample and is possibly enriched due to up-concentration of the purified PilG_RKK43-45AAA_ which was necessary because of low protein yields.
(TIF)

S8 Fig Example of DNA binding by PilG. EMSA performed with PilG_FL/1-410_ recombinant protein incubated with [γ^32^P] labeled dsDNA containing a DUS (0.1 nM HH7HH8, see Table 2). Samples with increasing protein concentrations [120, 140, 160, 180, 200, 220, 240, 250, 500, and 1000 nM], as indicated on top, were separated on a gel. Free DNA and the DNA-protein complex are indicated by arrows.
(TIF)

S9 Fig PilG co-purifies with the inner membrane. Separation of outer and inner membranes from *N*. *meningitidis* strain M1080 by sucrose gradient centrifugation. Samples from gradient fractions, taken from bottom to top and shown from left to right side, were separated by SDS-PAGE and stained with Coomassie Blue (top panel) and analyzed by immunoblotting, using antibodies against PilG, PilP, PilQ and PilW (lower panels). The positions of the molecular size markers are shown in kDa on the left. Complex and monomer forms of PilQ are indicated on the right. The outer membrane (OM), having a higher density than the inner membrane (IM), is located in the lower part of the gradient.
(TIF)

S10 Fig Expression of *Neisseria meningitidis* strain MC58 PilE and PilG proteins monitored by immunoblotting. (A) Western blot of pilus preparations with anti-PilE antiserum. (B) Western blot of whole cell lysates with anti-PilG antiserum. Sampled clones and protein amount per lane are indicated on top. MC58 wild type (WT), MC58 ΔpilG (ΔG), MC58-pilG:aph (aph), MC58-pilG-EEE:aph (EEE), and MC58-pilG-RKK:aph (RKK) are shown. Arrows indicate (A) PilE and (B) PilG specific bands. The positions of the molecular size markers are shown in kDa on the left.
(TIF)

S1 Table Oligonucleotides. Primers employed in PilG recombinant protein construction and alanine substitution mutants.
(DOCX)

S2 Table Recombinant proteins. Expression and purification of PilG recombinant proteins. All constructs contain a C-terminal 6×His-tag predicted to be located in the cytoplasm.
(DOCX)
