# Supplementary figures and images for: The Inner Membrane Protein PilG Interacts with DNA and the Secretin PilQ in Transformation
Source: PLoS One. 2015 Aug 6;10(8):e0134954. doi: 10.1371/journal.pone.0134954 (PMC4527729; doi:10.1371/journal.pone.0134954)

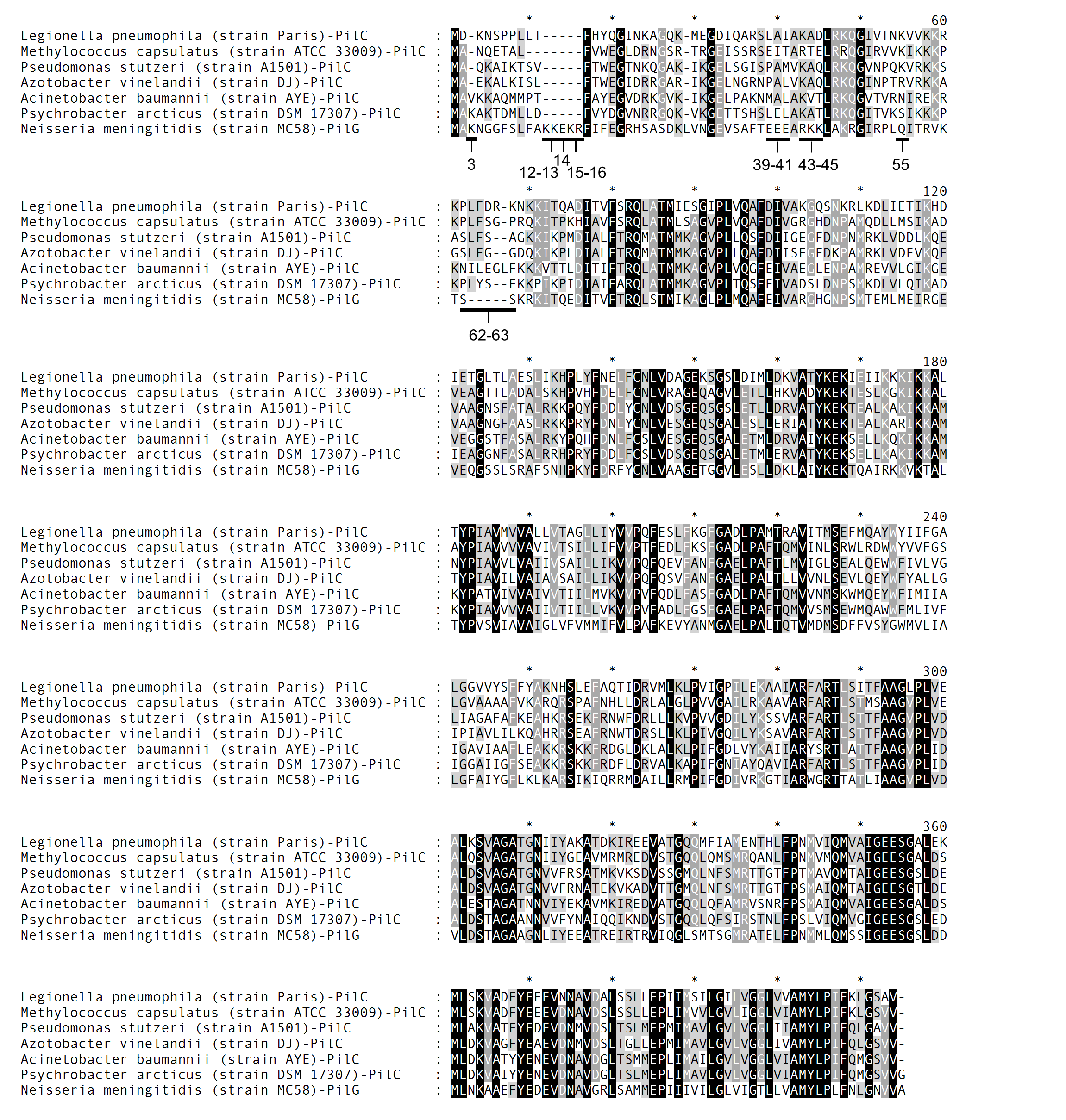

Supplement: S1 Fig — The full amino acid sequences of PilG orthologs from neisserial species and other competent bacteria were aligned using Clustal. Amino acids for alanine substitution-mutation, which were made in the progress of this study, are underlined and numbered. (TIF) [file pone.0134954.s001.TIF]

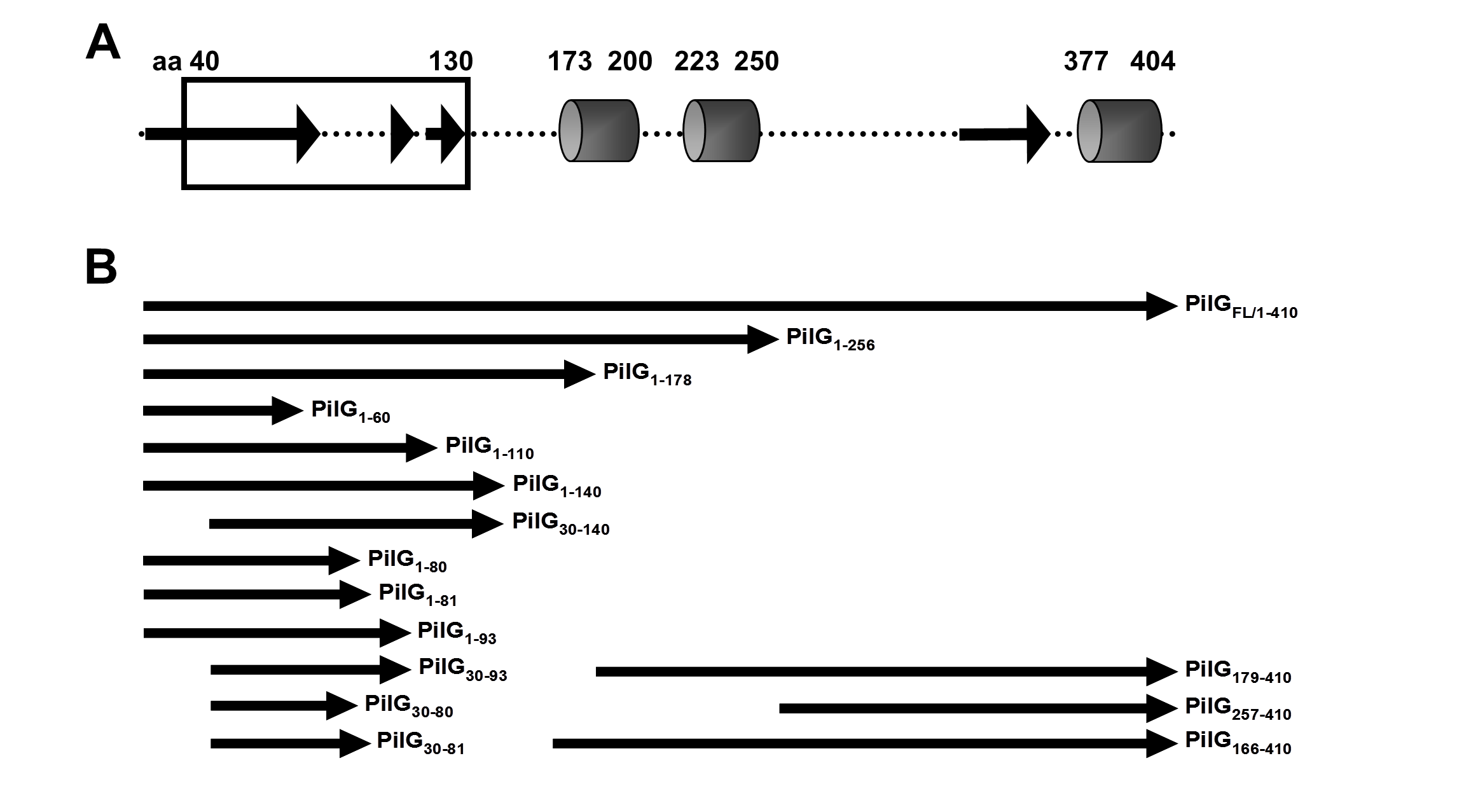

Supplement: S2 Fig — (A) Structural features as presented in Fig 1A. (B) All PilG constructs in position related to A with the names indicated to the right. All constructs contain an additional C-terminal 6×His-tag. (TIF) [file pone.0134954.s002.TIF]

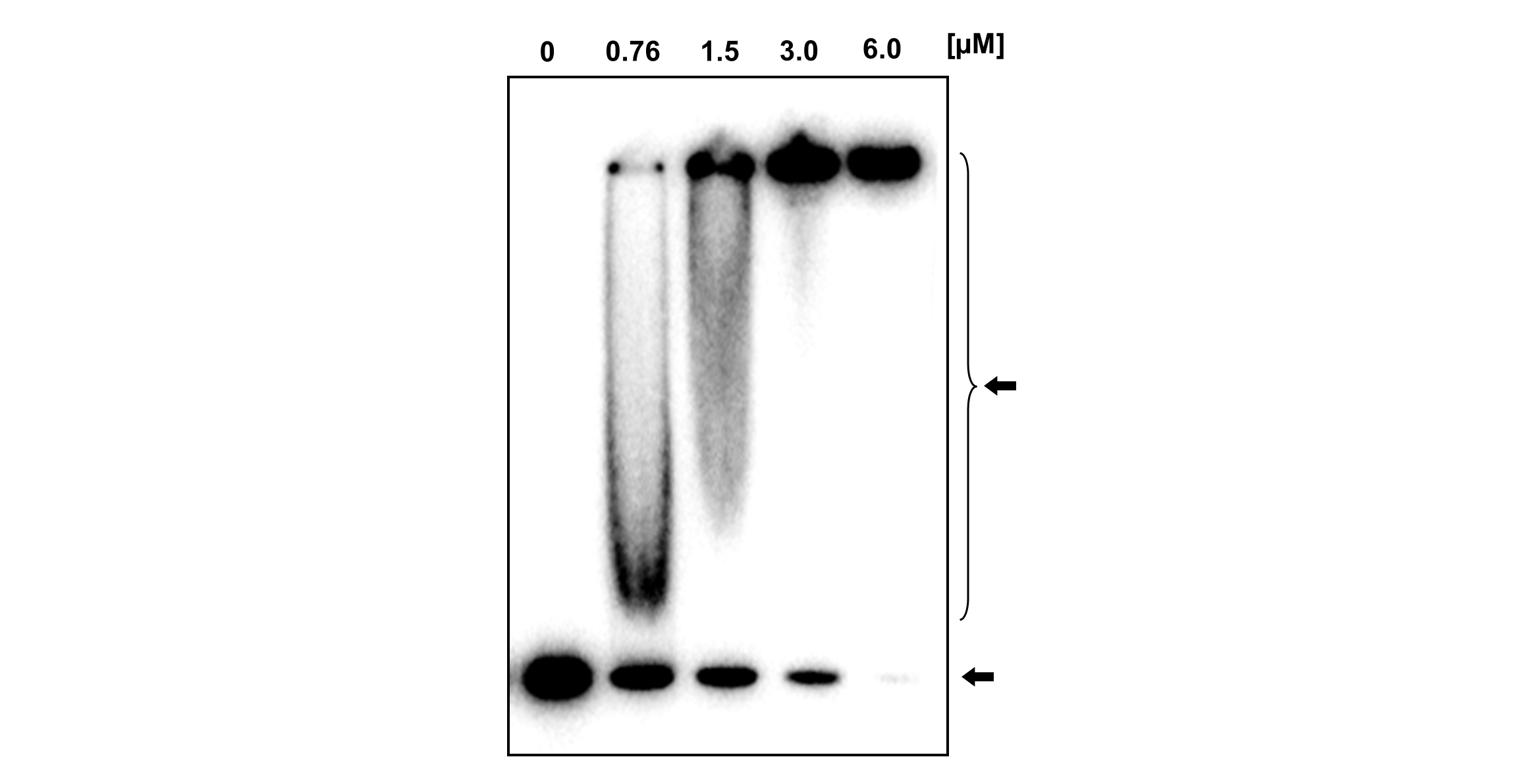

Supplement: S3 Fig — EMSA was performed on PilGFL recombinant protein incubated with a [γ32P]ATP labeled 52 bp dsDNA DUS containing substrate (HH7HH8) in the phosphate buffer system described in the methods part. For the controls see S4 Fig Based on three experiments the estimated concentrations required for half-maximal binding activity was 0.3 μM. Protein concentrations are given on top of the lanes in [μM]. The positions for the free DNA and the DNA-PilGFL complex are indicated by arrows. (TIF) [file pone.0134954.s003.TIF]

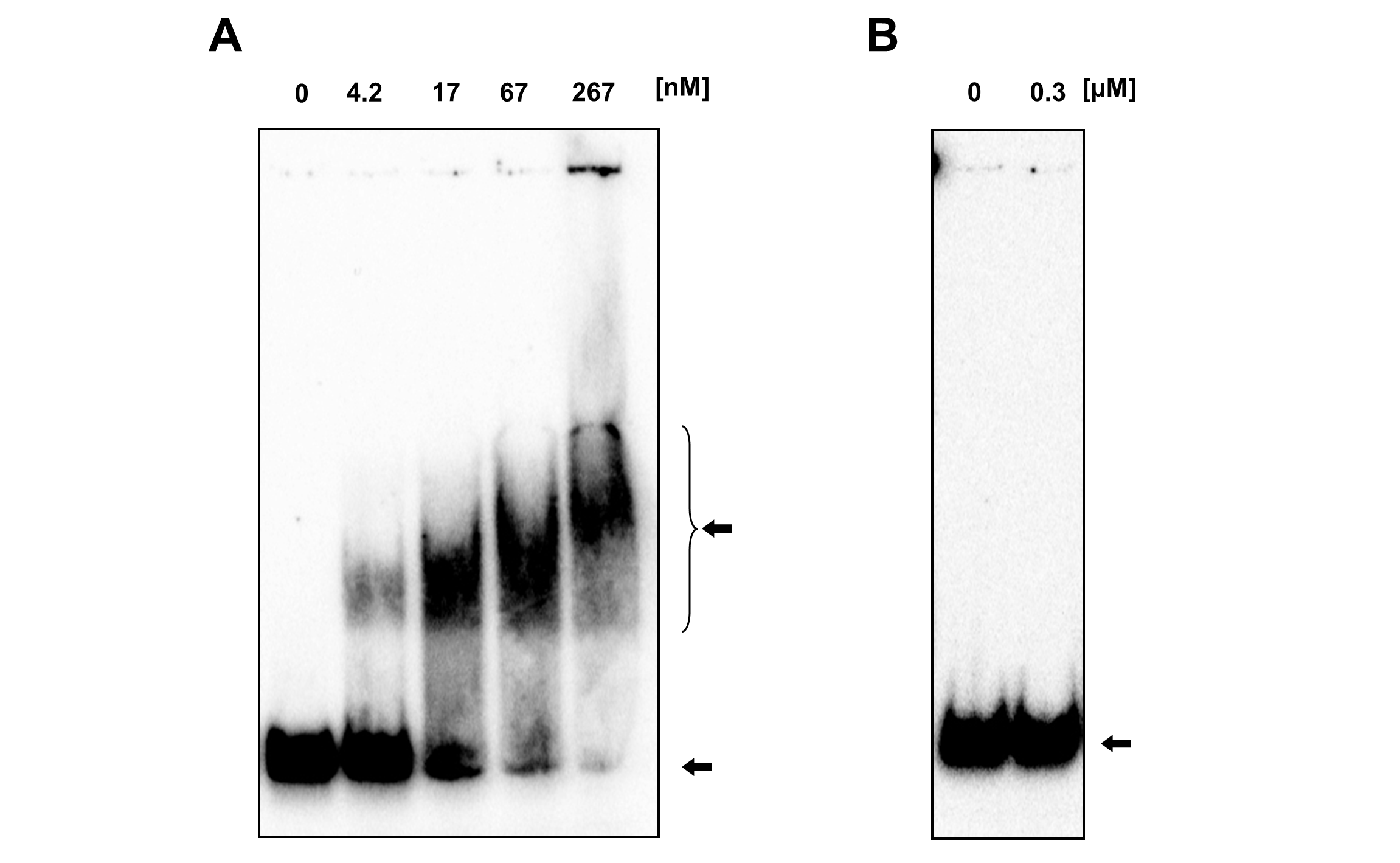

Supplement: S4 Fig — EMSA was performed with (A) the positive control protein Fpg and (B) with BSA as negative control. On top the lanes the concentrations of protein used are indicated, [nM] for Fpg and [μM] for BSA. The radiolabeled DNA used was double-stranded and without DUS. Positions for the free DNA and the DNA-Fpg complex are indicated by arrows. (TIF) [file pone.0134954.s004.TIF]

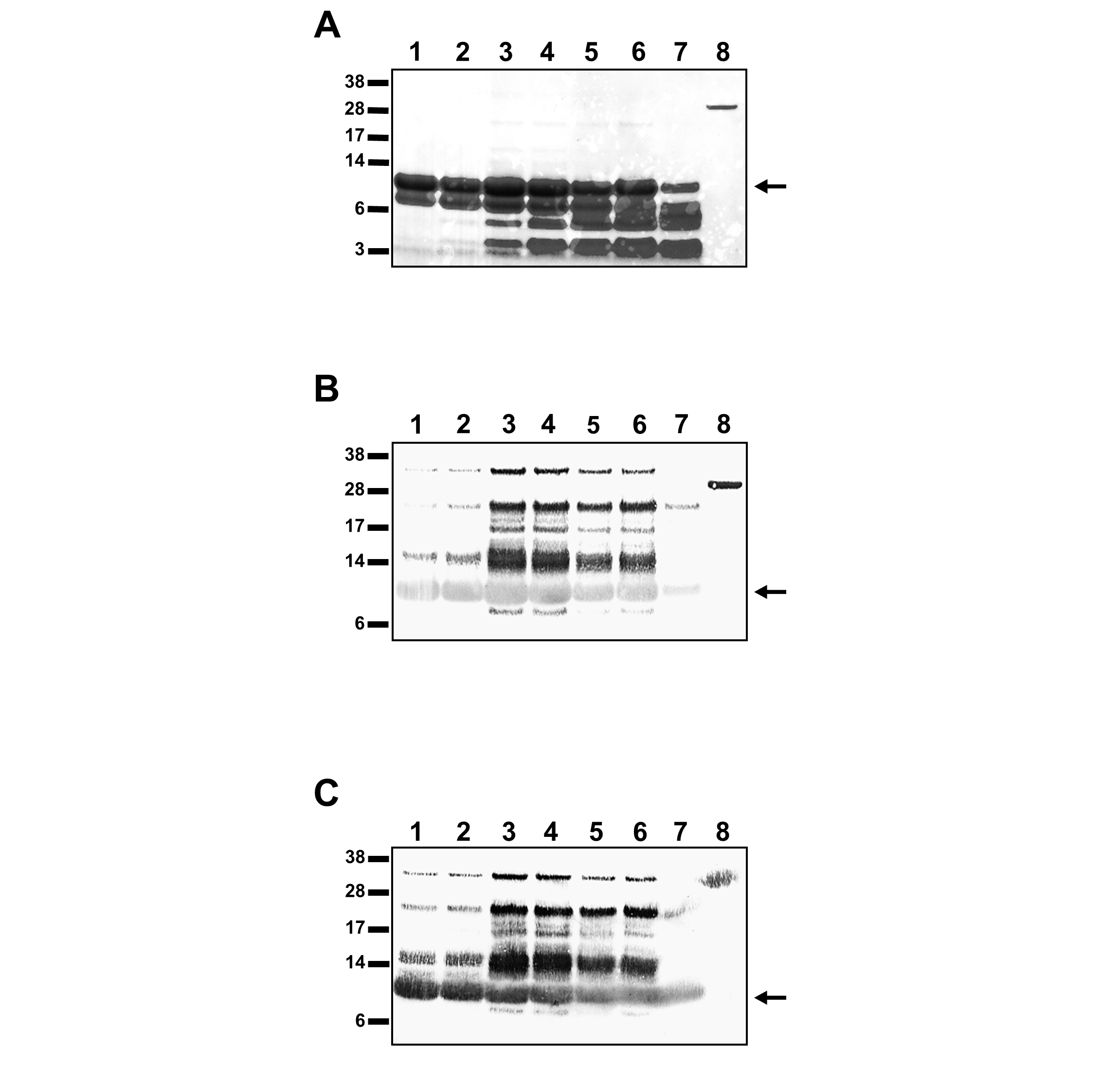

Supplement: S5 Fig — (A) Coomassie Blue-stained gel of endoproteinase cleaved PilG1-80. The DNA binding activity of the cleavage products was assessed with a solid phase overlay assay using 10 bp DUS+ (B) ssDNA and (C) dsDNA as substrates. Lanes 1 to 7 contain samples after different digestion times (1, 15 min; 2, 30 min; 3, 1 h; 4, 2 h; 5, 4 h; 6, 8 h; 7, 24 h, respectively) and lane 8 contains the positive control, Fpg. The positions of the molecular size markers are shown in kDa on the left. The arrow indicates the full-length protein. (TIF) [file pone.0134954.s005.TIF]

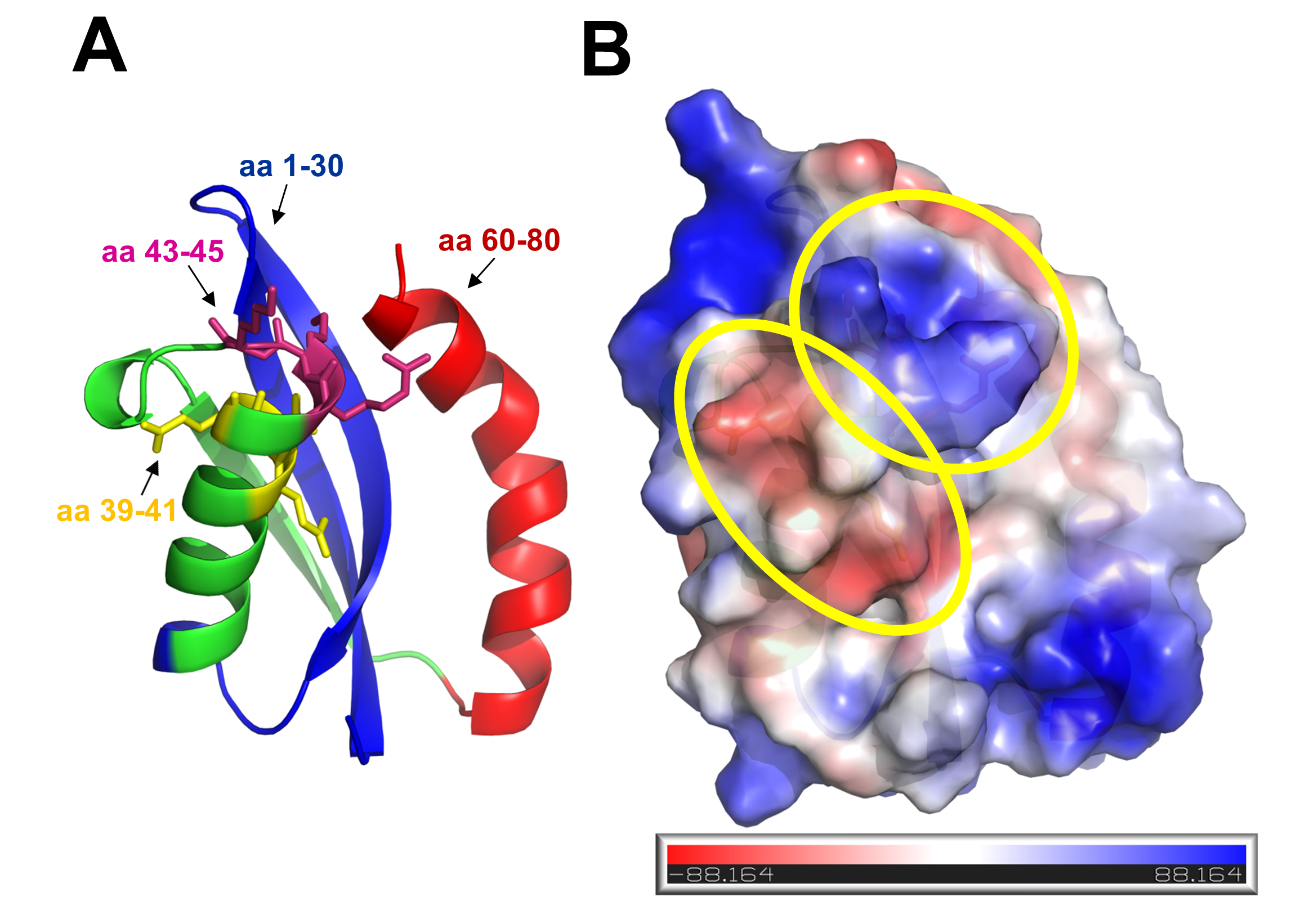

Supplement: S6 Fig — The (B) electrostatic charge on the protein surface of (A) the predicted structure for the N-terminal PilG (aa 1–80) was computed using PyMol. (B) The negative charged area (coloured in red) due to the amino acids 39–41 EEE and the positive charged area (coloured in blue) due to the amino acids 43–45 RKK are encircled. (TIF) [file pone.0134954.s006.TIF]

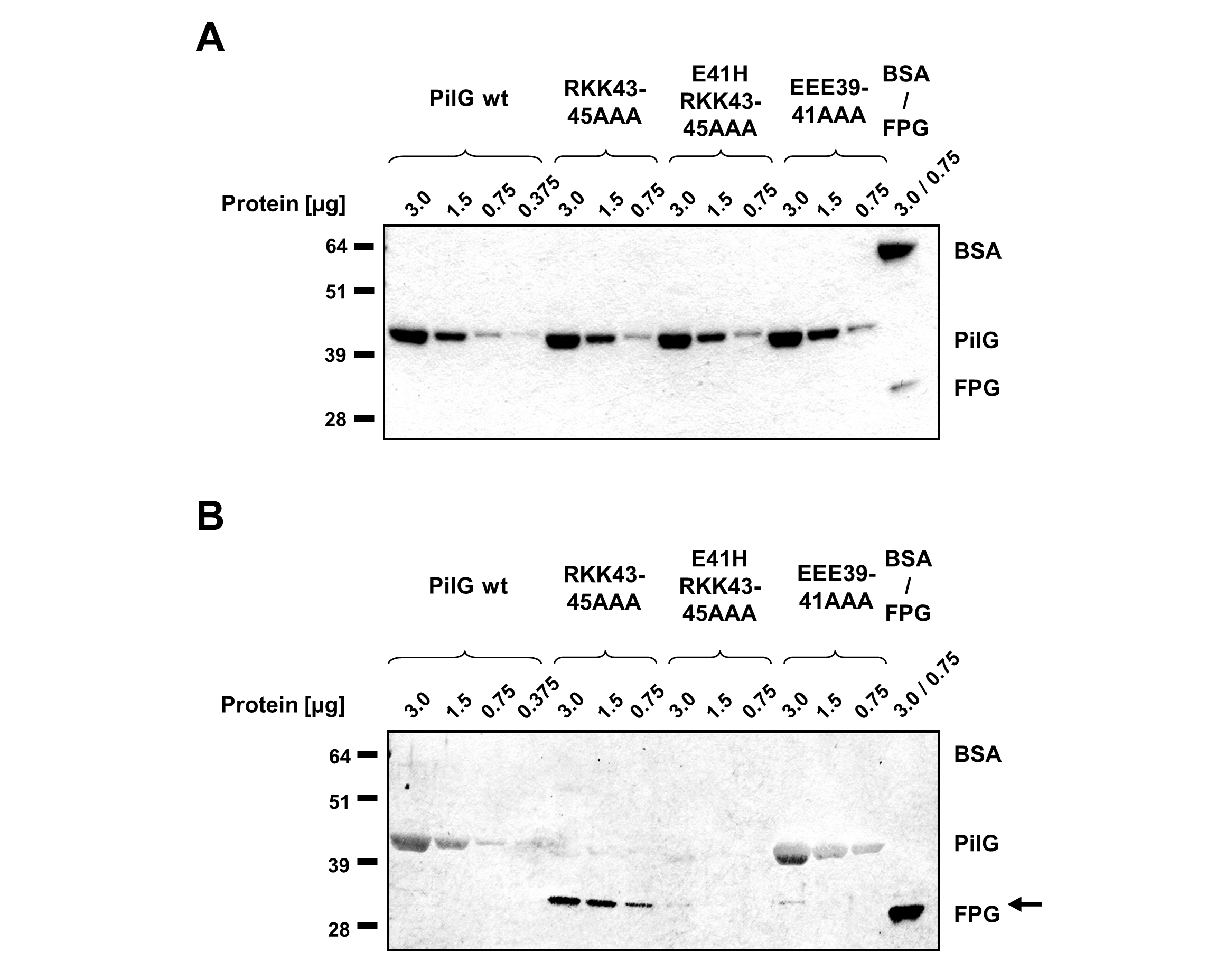

Supplement: S7 Fig — (A) Coomassie Blue-staining confirms equal amounts of protein loaded for the assay shown in panel B. (B) A solid phase overlay assay for protein-DNA interaction shows the DNA binding activity of PilGFL compared to the PilG alanine substitution mutants PilGRKK43-45AAA, PilGE41H/RKK43-45AAA and PilGEEE39-41AAA. The DNA substrate used was double-stranded containing AT-DUS. Fpg and BSA were used as positive and negative controls, respectively. PilGRKK43-45AAA and PilGE41H/RKK43-45AAA showed reduced DNA binding compared to PilGFL and PilGEEE39-41AAA. The positions of the molecular size markers are shown in kDa on the left. Protein amounts are given on top of the lanes. The arrow indicates an unknown contaminant that is not visible by protein staining in the PilGRKK43-45AAA sample and is possibly enriched due to up-concentration of the purified PilGRKK43-45AAA which was necessary because of low protein yields. (TIF) [file pone.0134954.s007.TIF]

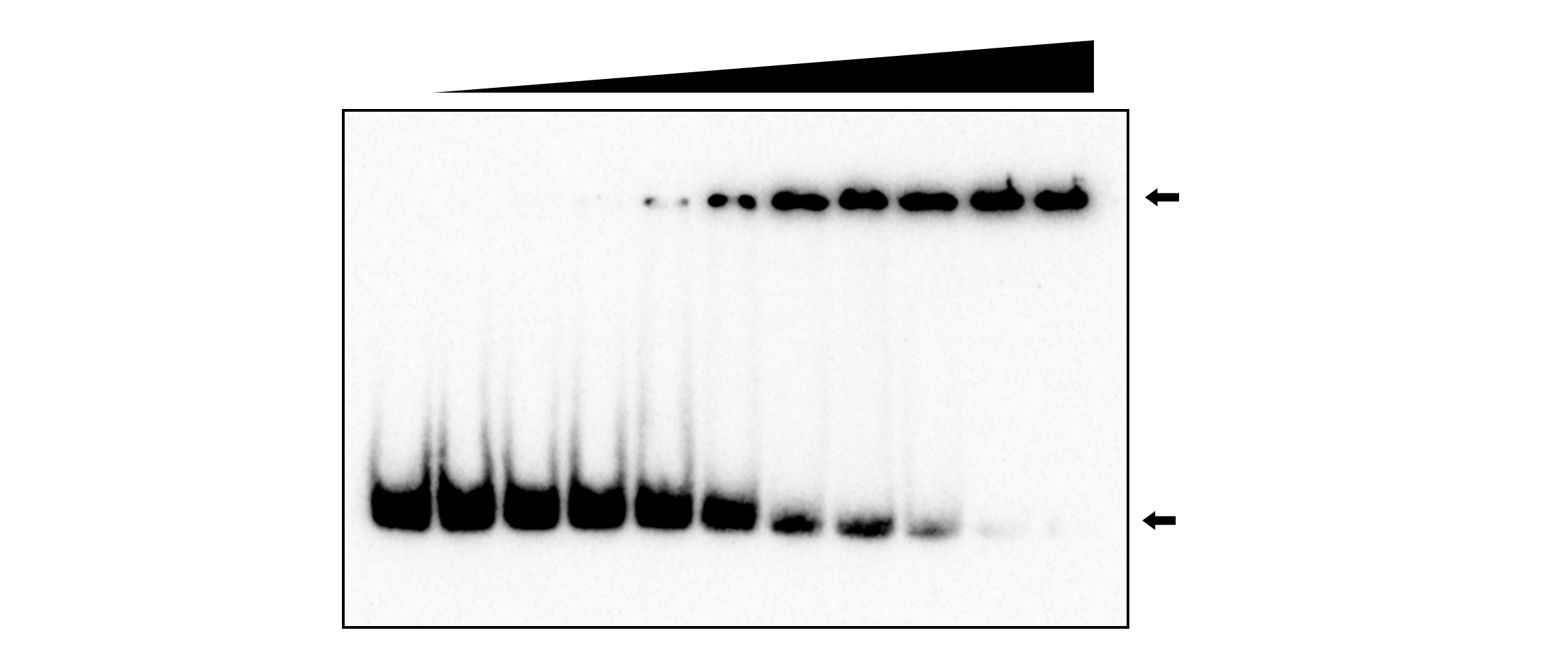

Supplement: S8 Fig — EMSA performed with PilGFL/1-410 recombinant protein incubated with [γ32P] labeled dsDNA containing a DUS (0.1 nM HH7HH8, see Table 2). Samples with increasing protein concentrations [120, 140, 160, 180, 200, 220, 240, 250, 500, and 1000 nM], as indicated on top, were separated on a gel. Free DNA and the DNA-protein complex are indicated by arrows. (TIF) [file pone.0134954.s008.TIF]

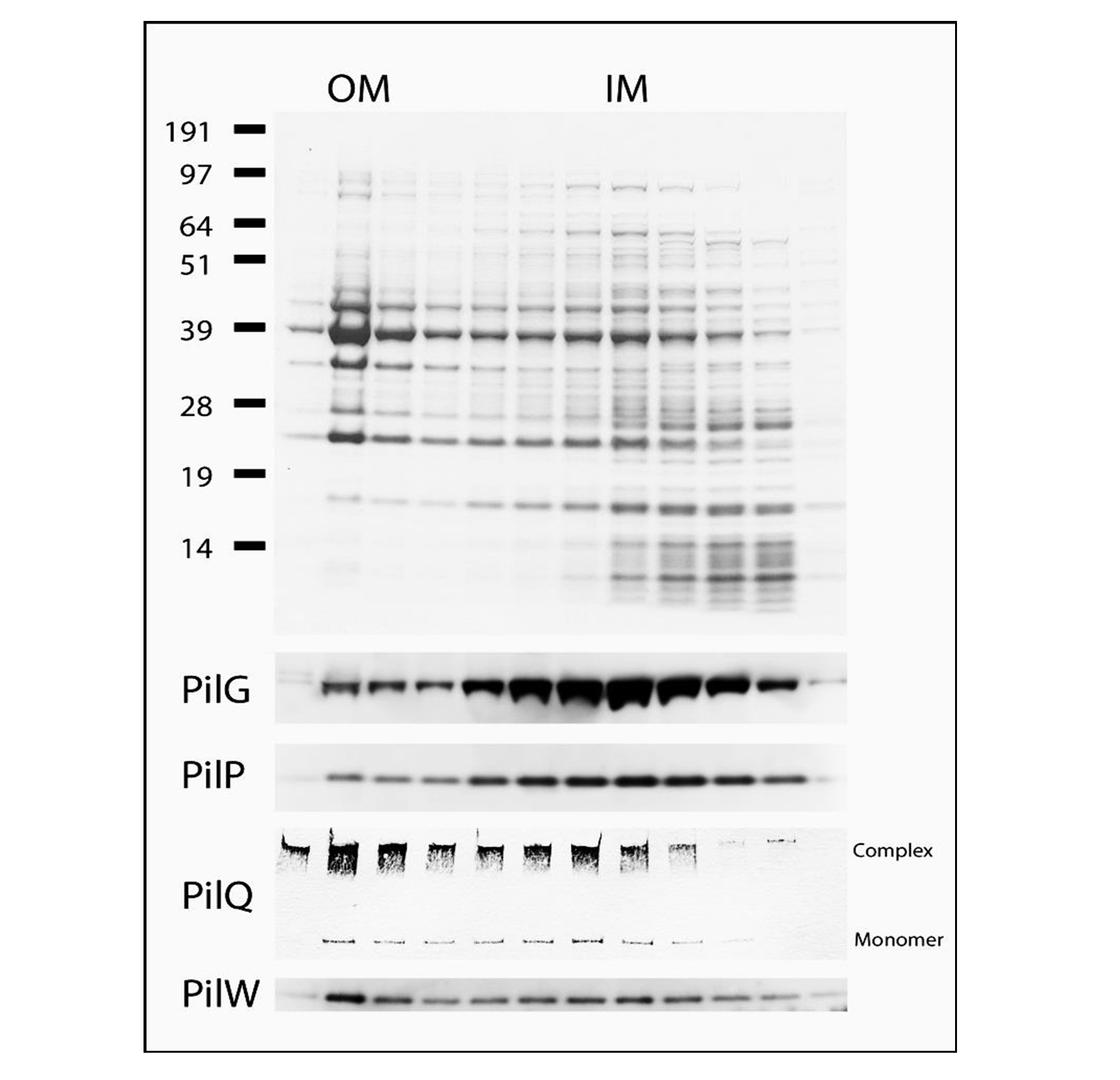

Supplement: S9 Fig — Separation of outer and inner membranes from N. meningitidis strain M1080 by sucrose gradient centrifugation. Samples from gradient fractions, taken from bottom to top and shown from left to right side, were separated by SDS-PAGE and stained with Coomassie Blue (top panel) and analyzed by immunoblotting, using antibodies against PilG, PilP, PilQ and PilW (lower panels). The positions of the molecular size markers are shown in kDa on the left. Complex and monomer forms of PilQ are indicated on the right. The outer membrane (OM), having a higher density than the inner membrane (IM), is located in the lower part of the gradient. (TIF) [file pone.0134954.s009.TIF]

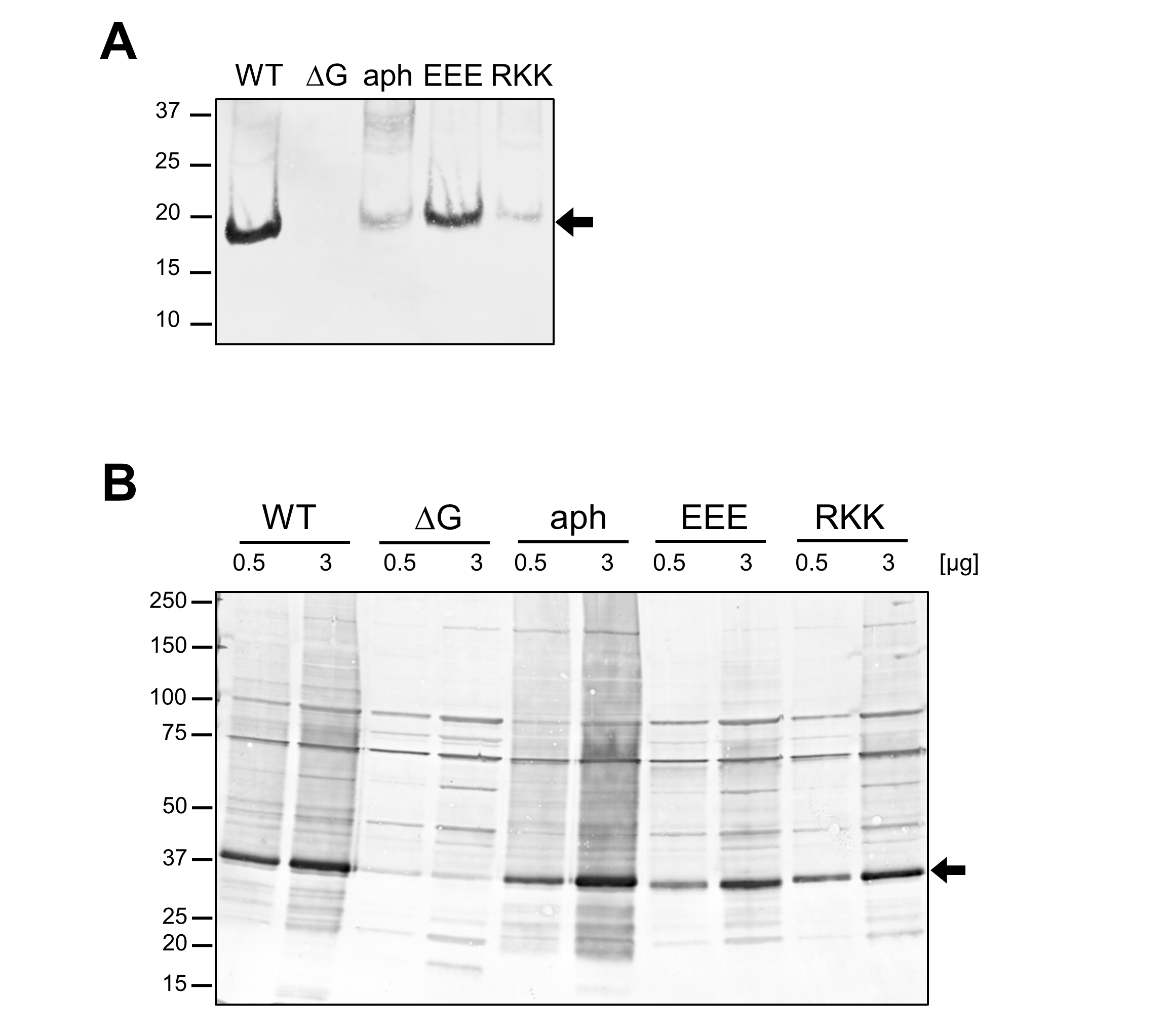

Supplement: S10 Fig — (A) Western blot of pilus preparations with anti-PilE antiserum. (B) Western blot of whole cell lysates with anti-PilG antiserum. Sampled clones and protein amount per lane are indicated on top. MC58 wild type (WT), MC58 ΔpilG (ΔG), MC58-pilG:aph (aph), MC58-pilG-EEE:aph (EEE), and MC58-pilG-RKK:aph (RKK) are shown. Arrows indicate (A) PilE and (B) PilG specific bands. The positions of the molecular size markers are shown in kDa on the left. (TIF) [file pone.0134954.s010.TIF]
