# Supplementary material for: The Inner Membrane Protein PilG Interacts with DNA and the Secretin PilQ in Transformation
Source: PLoS One. 2015 Aug 6;10(8):e0134954. doi: 10.1371/journal.pone.0134954 (PMC4527729; doi:10.1371/journal.pone.0134954)
Supplement: S1 Table — Primers employed in PilG recombinant protein construction and alanine substitution mutants. (DOCX) [file pone.0134954.s011.docx]

**S1 Table. Oligonucleotides.** Primers employed in PilG recombinant protein construction and alanine substitution mutants.

| **Primer** | **Sequence (5’→3’)^*^** | **RE site** |
| --- | --- | --- |
| **PilG full-length / partial constructs** | | |
| SVB66 | CGTCTAGAAATAATTTTGTTTAACTTTAAGAAGGAGATATACCATGGCTAAAAACGGAGGA | *Xba*I |
| SVB67 | GCCTCGAGGGCGACCACGTTGCCCAAAT | *Xho*I |
| SF76 | CGCCATGGCCATCCGCAAAAAGGTAAAAACC | *Nco*I |
| SAF-Tn5-aph-for | gcggatccTAGACTGGGCGGTTTTATGG | *Bam*HI |
| OHA_AphEcoRI_REV | gcgaattcTCATTTCGAACCCCAGAGTC | *Eco*RI |
| SF135 | gcggatccaggatattgttgcgggtcaa | *Bam*HI |
| SF136 | gcaagctTTCAGACGGCatacgaaccgtcgtccaaac | *Hind*III |
| OHA66 | GCCTCGAGacgccgttggattttaatcgaacgcgcctt | *Xho*I |
| OHA67 | GCCTCGAGcggataggtcagtgcggtt | *Xho*I |
| OHA68 | GACCATGGTATCCGTCATCGCCGTCGCCA | *Nco*I |
| OHA69 | GACCATGGatgccatactgctgcgta | *Nco*I |
| EL001 | GCCTCGAGTTTCACACGGGTAATCTGCAA | *Xho*I |
| EL002 | GCCTCGAGCATCAGCATTTCCGTCATAGA | *Xho*I |
| EL003 | GCCTCGAGCAGATTGCAGTAGAAGCGGT | *Xho*I |
| EL004-1 | GCCTCGAGGGACAGCTGGCGGGTGAA | *Xho*I |
| EL004-2 | GCCTCGAGCGTGGACAGCTGGCGGGT | *Xho*I |
| EL005 | GCCTCGAGAAATGCCTGCATCAGCGGCAG | *Xho*I |
| EL006 | CGTCTAGAAATAATTTTGTTTAACTTTAAGAAGGAGATATACCATGAACGGCGAAGTATCCGCGTTT | *Xba*I |
| **Alanine substitution mutants ^†^** | | |
| K3A | CGCCATGGAT**GCA**AACGGAGGATTT | *Nco*I |
| KK12-13AA | CGCCATGGCTAAAAACGGAGGATTTTCTTTGTTCGCA**GCGGCA**GAAAAACGCTTTATC | *Nco*I |
| E14A | CGCCATGGCTAAAAACGGAGGATTTTCTTTGTTCGCAAAGAAA**GCA**AAACGCTTTATCTT | *Nco*I |
| KR15-16AA | CGCCATGGCTAAAAACGGAGGATTTTCTTTGTTCGCAAAGAAAGAA**GCAGCC**TTTATCTTTGAA | *Nco*I |
| EEE39-41AAA forward | CGTTTACC**GCAGCAGCG**GCGCGCAAAAAGCTTGCAAAACGCGGCATCC | *Hind*III |
| EEE39-41AAA reverse | CGTTTTGCAAGCTTTTTGCGCGC**CGCTGCTGC**GGTAAACGCGGATACTTCG | *Hind*III |
| RKK43-45AAA forward | GAGGCG**GCCGCAGCA**CTGGCAAAACGCGGAATTCGCCCGTTGCAG | *Eco*RI |
| RKK43-45AAA reverse | GGCGAATTCCGCGTTTTGCCAG**TGCTGCGGC**CGCCTCTTCTTCGG | *Eco*RI |
| Q55A forward | CGCGGAATTCGCCCGTTG**GCG**ATTACCCGTGTGAAAACAAGC | *Eco*RI |
| Q55A reverse | CACACGGGTAAT**CGC**CAACGGGCGAATTCCGCGTTTTGCCAG | *Eco*RI |
| SS62-63AA forward | GCCCGCTGCAGATTACCCGTGTGAAA**GCAGCCGCC**AAGCGCAAAATCAC | *Pst*I |
| SS62-63AA reverse | CGCTT**GGCGGCTGC**TTTCACACGGGTAATCTGCAGCGGGCGGATGCCGC | *Pst*I |

^*^ Restriction sites are underlined.
^†^ Changed bases leading to amino acid substitutions are indicated in bold.
