# Supplementary material for: The Inner Membrane Protein PilG Interacts with DNA and the Secretin PilQ in Transformation
Source: PLoS One. 2015 Aug 6;10(8):e0134954. doi: 10.1371/journal.pone.0134954 (PMC4527729; doi:10.1371/journal.pone.0134954)
Supplement: S2 Table — Expression and purification of PilG recombinant proteins. All constructs contain a C-terminal 6×His-tag predicted to be located in the cytoplasm. (DOCX) [file pone.0134954.s012.docx]

**S2 Table. Recombinant proteins.** Expression and purification of PilG recombinant proteins. All constructs contain a C-terminal 6× His-tag predicted to be located in the cytoplasm.

| **Construct [included amino acids]** | **Trans-membrane helices (TMH) (predicted)** | **Expression** | **Solubility and purification** |
| --- | --- | --- | --- |
| 1-60 | 0 | Yes | soluble without DDM^†^ |
| 1-80 | 0 | Yes | soluble without DDM |
| 1-81 | 0 | Yes | soluble without DDM |
| 30-80 | 0 | Yes | isolated as inclusion bodies and purified using urea |
| 30-81 | 0 | Yes | isolated as inclusion bodies and purified using urea |
| 1-93 | 0 | No^‡^ |  |
| 30-93 | 0 | No |  |
| 1-110 | 0 | No |  |
| 1-140 | 0 | Yes | soluble in DDM although lacking TMH |
| 30-140 | 0 | No |  |
| 1-178 | 0 | Yes | soluble in DDM although lacking TMH |
| 1-256 | 2 | Yes | soluble in DDM |
| 257-410 | 2 | Yes | soluble in DDM and lysozyme, unstable |
| 179-410 | 4 | No |  |
| 166-410 | 4 | Yes | soluble in DDM |
| FL / 1-410 | 4 | Yes | soluble in DDM |

^†^ DDM = n-Dodecyl β-D-maltoside
^‡^ No = no detection with anti-His-tag or anti-PilG antibodies
